# Synthesis, molecular docking, ADMET studies and biological evaluation of fused pyrazolopyridopyrimidine derivatives as antioxidant and antimicrobial agents

**DOI:** 10.1038/s41598-025-30217-9

**Published:** 2025-12-11

**Authors:** Hemat S. Khalaf, Ahmed F. El-Sayed, Ashraf A. Sediek, Mohamed A. A. Radwan

**Affiliations:** 1 Department of Photochemistry, Chemical Industries Research Institute, National Research Centre, Dokki, 12622 Cairo Egypt; 2 Microbial Genetics Department, Biotechnology Research Institute, National Research Centre, Giza, Egypt; 3 Egypt Center for Research and Regenerative Medicine (ECRRM), Cairo, Egypt; 4 Chemical Industries Institute, National Research Centre, Dokki, Cairo, 12622 Egypt; 5 Applied Organic Chemistry Department, National Research Centre, Dokki, Cairo, 12622 Egypt

**Keywords:** Pyrazolopyridine, Pyrazolopyridopyrimidine, Antimicrobial, Antioxidant, Molecular docking, ADMET, Biochemistry, Chemistry, Drug discovery, Microbiology

## Abstract

This study describes the design, synthesis, and biological evaluation of fused pyrazolopyridopyrimidine analogues as potential antimicrobial and antioxidant agents. The chemical structures of the newly synthesized compounds were confirmed by elemental analyses and spectroscopic methods. The compounds were screened for antimicrobial activity against Escherichia coli ATCC 25,915, Staphylococcus aureus ATCC 25,923, Enterococcus faecalis ATCC 29,212, Pseudomonas aeruginosa ATCC 10,145, and Streptococcus mutans ATCC 25,175. Compound 7 exhibited pronounced inhibitory effects. Evaluation of antioxidant potential using the DPPH radical scavenging assay revealed that compound 15 displayed superior activity, with inhibition values of 44.44 ± 0.80%, 16.50 ± 0.90%, and 0.0% at concentrations of 2.0, 1.0, and 0.5 mg/mL, respectively. To elucidate the molecular basis of these activities, docking studies were performed, which demonstrated favorable binding interactions with key microbial and antioxidant-related targets. Furthermore, ADMET profiling and drug-likeness assessments confirmed that compounds 7, 9, 11, and 15 adhere to Lipinski’s Rule of Five, suggesting favorable oral bioavailability and promising pharmacokinetic properties. Taken together, these findings highlight fused pyrazolopyridopyrimidines as structurally promising scaffolds for the development of novel antimicrobial and antioxidant therapeutics, warranting further structural optimization and preclinical investigation.

**Supplementary Information:**

The online version contains supplementary material available at 10.1038/s41598-025-30217-9.

## Introduction

**Fig. 1:**
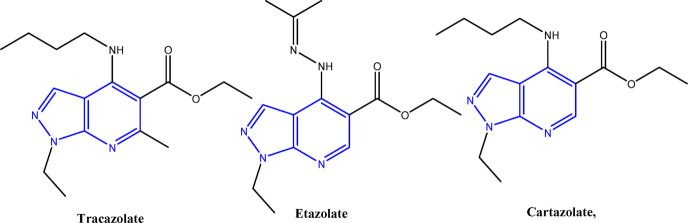
Drugs containing Pyrazolo[3,4-b]pyridine skeleton.

Nitrogen-containing heterocyclic are greatly effective structures for recognizing bioactive compounds in pharmaceuticals and agrochemicals^1–4^. Heterocyclic scaffolds containing the pyridine ring are related to multiple pharmacological effects, including antibacterial^5^, anticancer^6^, antiviral^7^, anticonvulsant^8^, antifungal^9^, and anti-HIV properties^10^. In agrochemical and pharmaceutical investigation, the pyrazolopyridine framework has appeared as a critical structural feature in an assortment of heterocyclic skeletons^11^. This skeleton is a common in medicinal chemistry owing to its wide-ranging of biological properties, including GSK-3 inhibition^12^, anticancer^13–15^, antimicrobial^16–18^, antileishmanial^19^, cyclin-dependent kinase inhibition^20^, anxiolytic^21^, anti-inflammatory^22^, cardiovascular^23^, anti-Alzheimer^24^, antiviral^25^, and antimalarial effects^26^. Moreover, pyrazolopyridines used as adenosine antagonists^27^, promote bone metabolism^28^, act as herbicide regulators^29^, and decrease platelet aggregation^30^. Investigations of marketed drugs incorporating the 1 H-pyrazolo[3,4-b]pyridine scaffold, including Tracazolate, Etazolate, and Cartazolate, have demonstrated their broad pharmacological applications, principally in treating anxiety linked to GABA-induced neuroinhibition (Fig. 1)^31^.

**Fig. 2:**
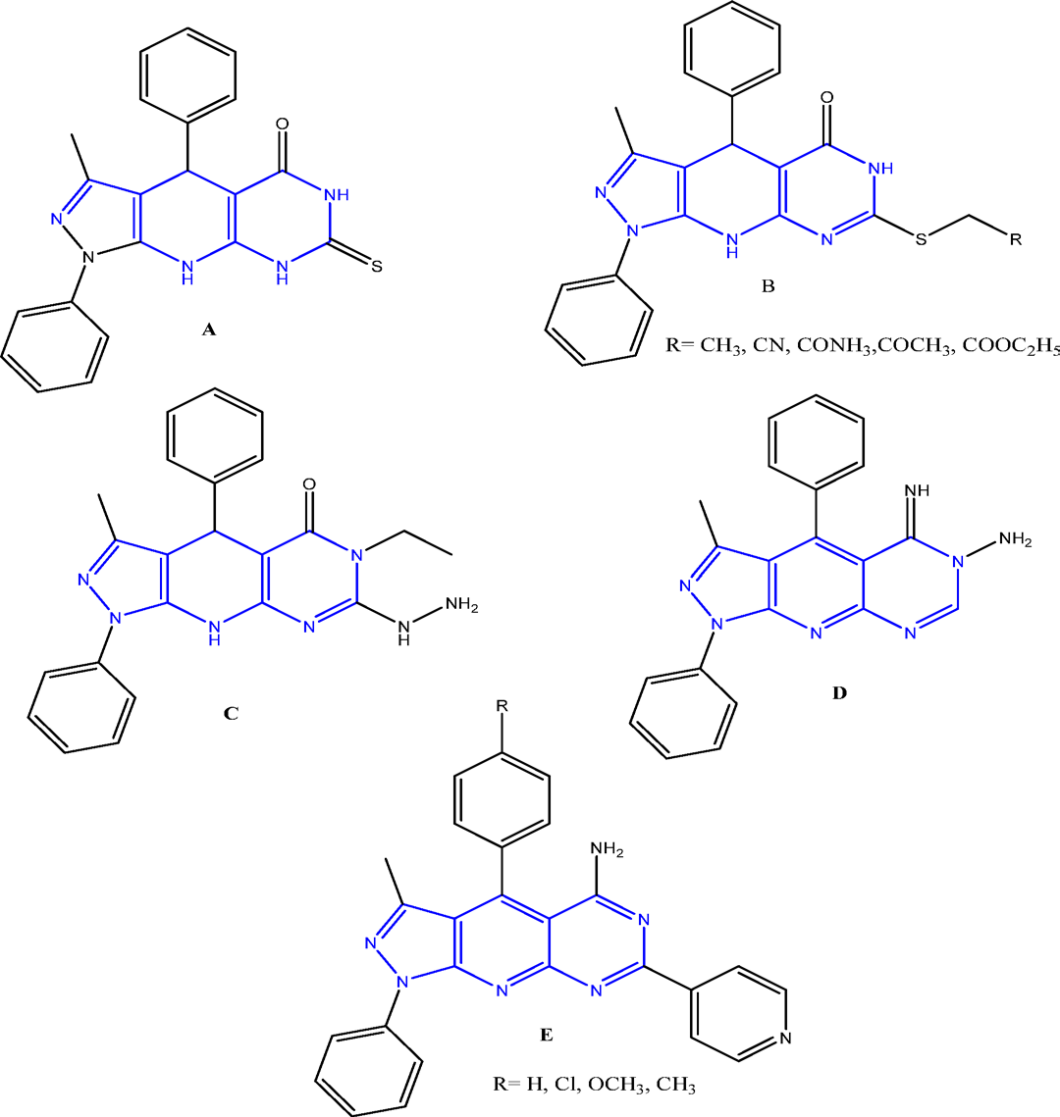
Bioactive pyrazolopyridopyrimidine derivatives.

A variety of synthetic methodologies have been reported for the preparation of pyrazolopyridines, permitting optimization under different reaction conditions^32,33^. Studies on fused pyrazolo[3,4-b]pyridine derivatives, with different isomers, have shown promising biological potential, supporting their improvement as new therapeutic agents. Among these, the pyrazolopyridopyrimidine framework is a fused heterocycle comprising pyrazole, pyridine, and pyrimidine rings, and constitutes an important class in medicinal chemistry due to its broad spectrum of biological activities. Interest in these fused systems has grown in response to the increasing request for bioactive pharmaceutical molecules. Pyrazolopyridopyrimidine derivatives have been recognized for their diverse pharmacological properties, including antibacterial^34–36^, antifungal^37^, antimicrobial^38,39^ and antitumor^40,41^ activities. For example, derivatives **A-C**^38^have exhibited broad-spectrum antimicrobial activity, while compound **D**^39^ revealed significant antibacterial potential. Furthermore, compounds **E**^38^ demonstrated antifungal activity (Fig. 2).

Recent studies highlight the role of pyrazolopyridopyrimidine derivatives in targeted therapy for diseases like cancer and infections^40^. Their unique electronic and steric properties let them to interact effectively with biological targets. Molecular docking studies and ADMET profiling are now common tools for discovering their drug-likeness^41^. In continuation of our research on the development of novel biologically active heterocycles, we report the synthesis and evaluation of new fused pyrazolopyridopyrimidine derivatives^42–46^. and in line with previous literature, we aim to synthesize fused pyrazolopyridopyrimidine compounds to develop more effective applicants.

## Results and discussion

### Chemistry

**Fig. 3:**
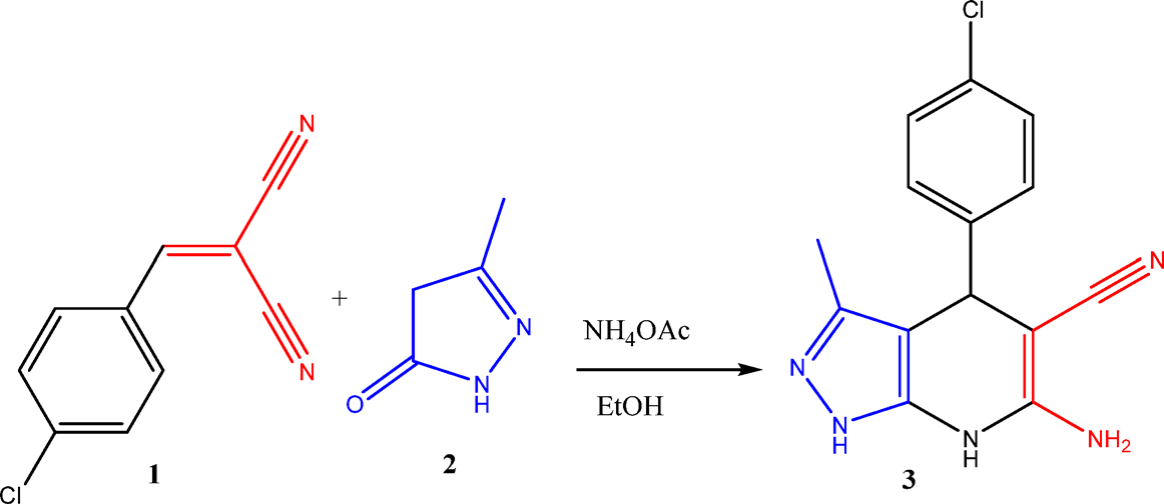
Synthesis of pyrazolopyridine deriivatives.

**Fig. 4:**
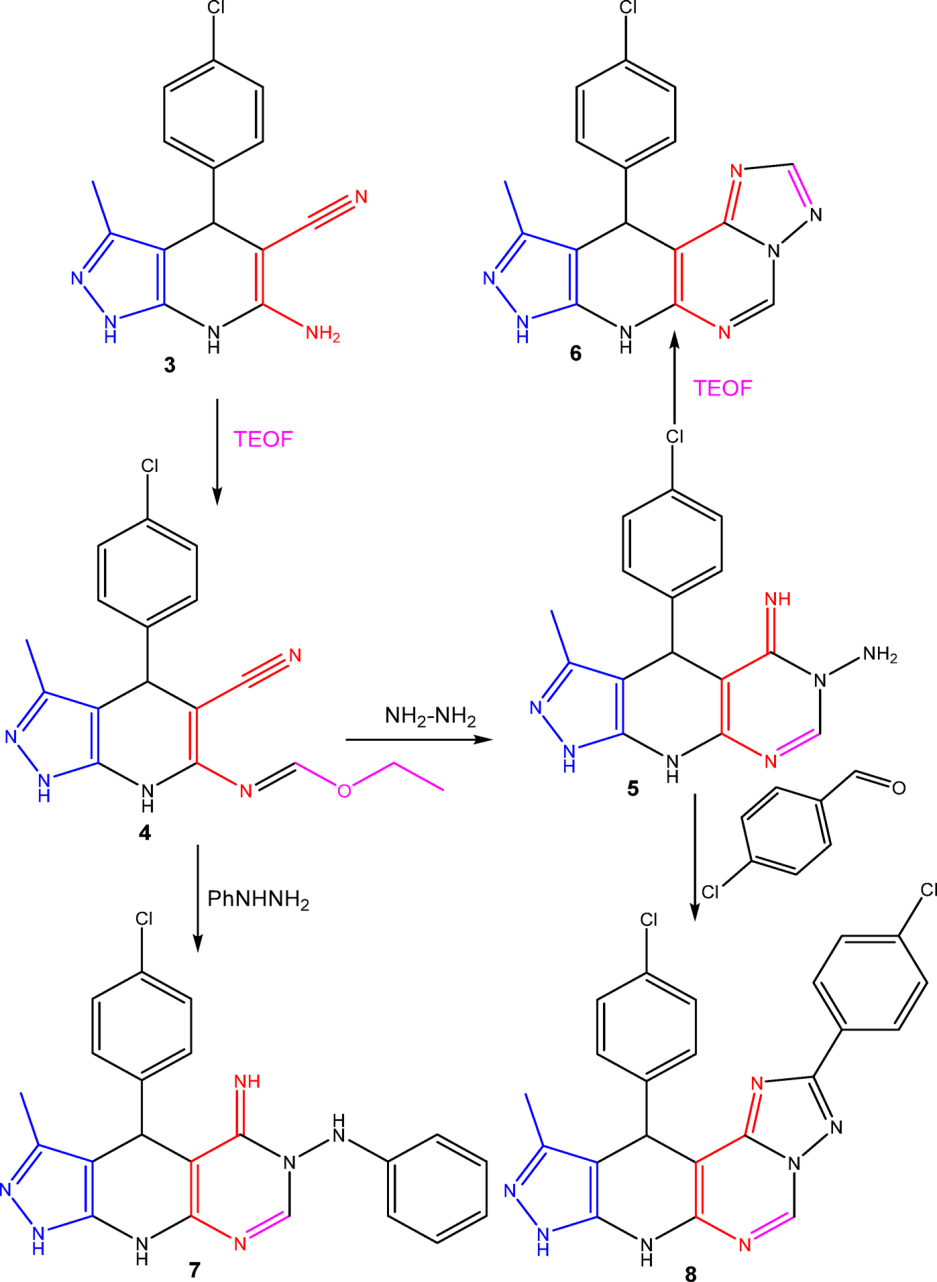
Synthesis of pyrazolopyridopyrimidine deriivatives (**6–8**).

**Fig. 5:**
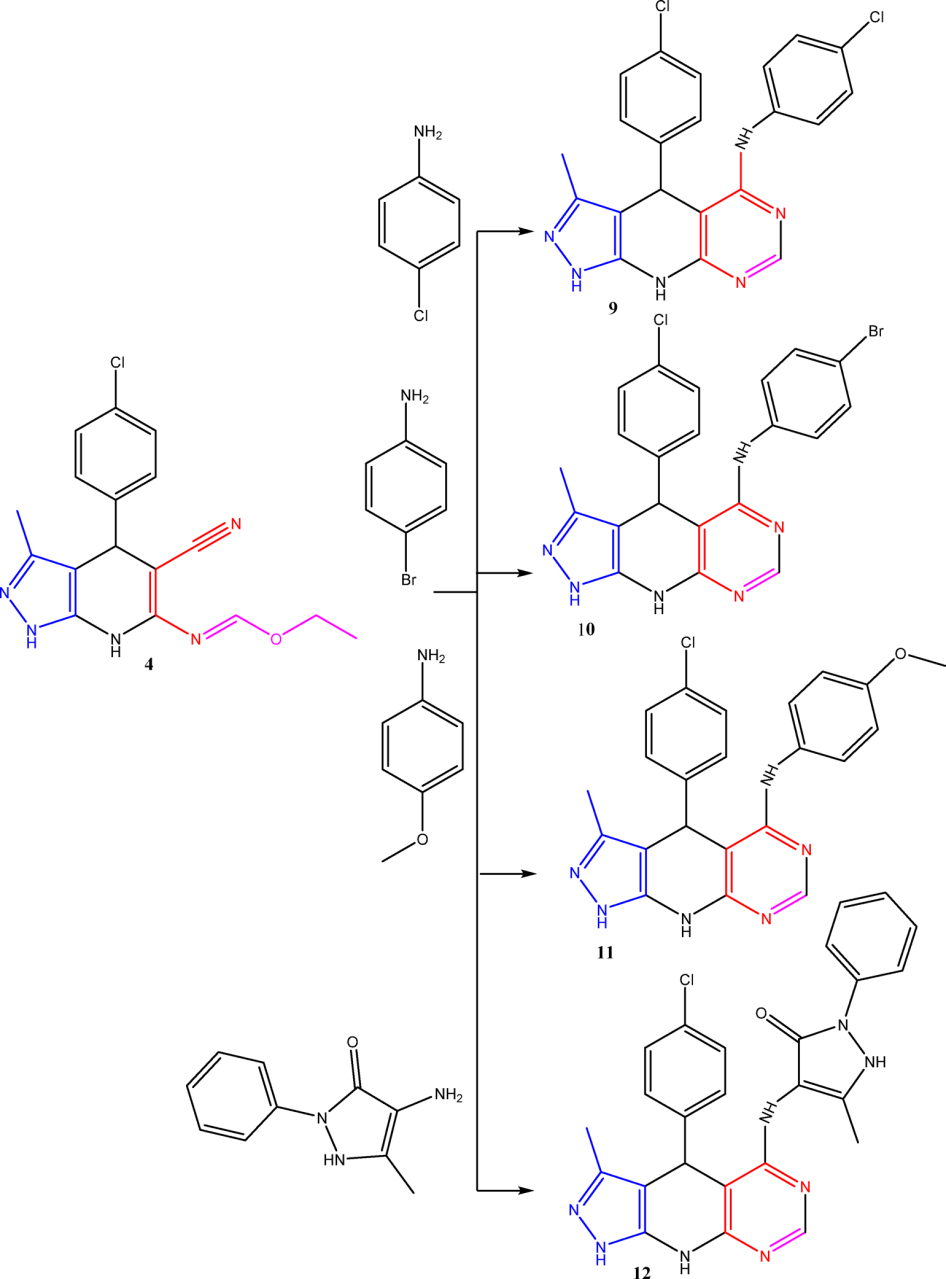
Synthesis of pyrazolopyridopyrimidine deriivatives (**9–12**).

**Fig. 6:**
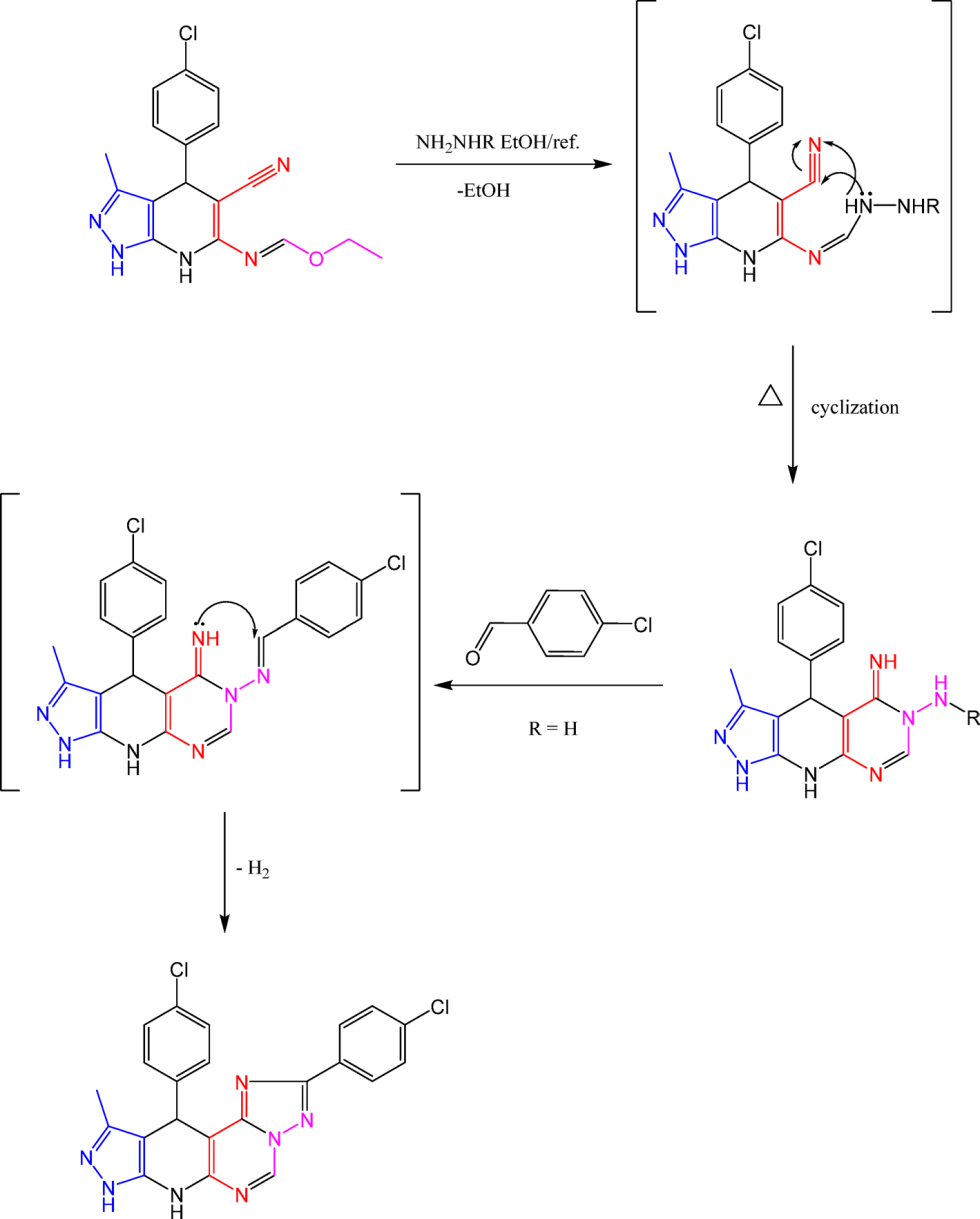
Plausible mechanism for the synthesis of pyrazolopyridopyrimidines.

The synthetic techniques for the newly synthesized fused pyrazolopyridopyrimidine analogues starting with the reported pyrazolopyridine derivative **3**^41^ are illustrated in Figs. 3, 4 and 5, and 6.

Figure 4 illustrates the synthetic pathway beginning with the condensation of compound **3** and triethylorthoformate, yielding the reported ethylformimidate **4**^47^. Following reaction of ethylformimidate **4** with hydrazine hydrate under reflux in ethanol produced 5-iminopyrazolopyridopyrimidine derivative **5**^47^. In turn, heating compound **5** with triethylorthoformate or formic acid in acetic anhydride led to the formation of new compound **6**. Additionally, refluxing compound **5** with 4-chlorobenzaldehyde in DMF produced pyrazolopyridotriazolopyrimidine derivative **8**. Also, the reaction between ethyl formimidate **4** and phenylhydrazine lead to the construction of pyrazolopyridopyrimidine derivative **7**.

Moreover, new pyrazolopyridopyrimidine derivatives (**9**–**12**) were synthesized through the reaction of ethyl formimidate **4** with various suitable amines under reflux conditions in acetic acid (Fig. 5). The plausible mechanism for the synthesis of pyrazolopyridopyrimidines (**8**) was illustrated in (Fig. 6).

**Fig. 7:**
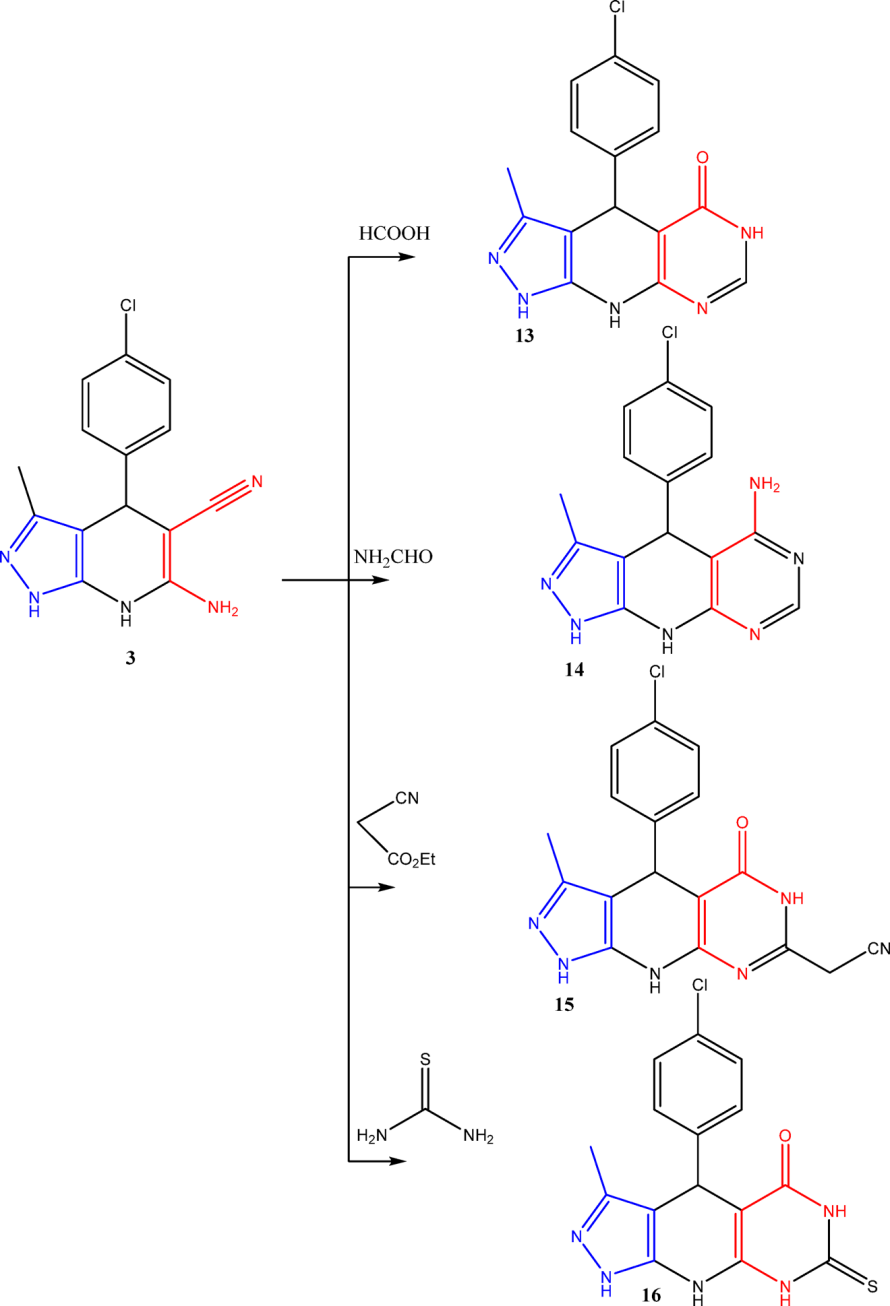
Synthesis of pyrazolopyridopyrimidine derivatives (**13–16**).

Finally, an additional pyrazolopyridopyrimidine derivatives (**13**–**16**) were synthesized by reacting compound **3** with formic acid, formamide, ethyl cyanoacetate, and thiourea, respectively, as outlined in (Fig. 7).

The newly synthesized pyrazolopyridopyrimidine derivatives were structurally clarified utilizing spectroscopic methods such as mass spectrometry, NMR, and infrared spectroscopy. (see experimental section).

### Antioxidant activities

**Fig. 8:**
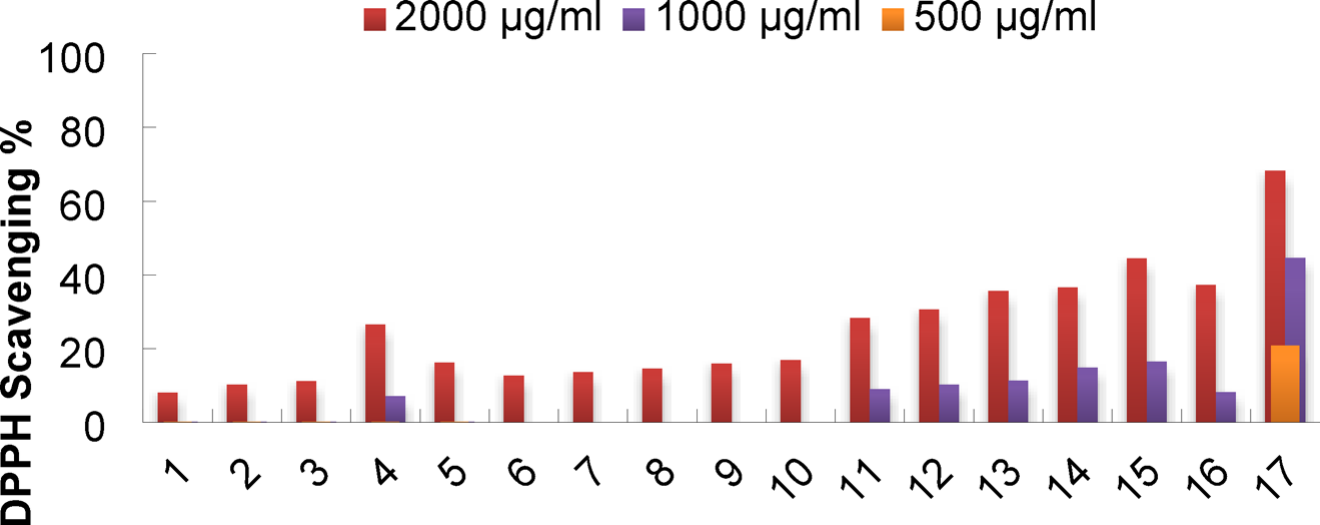
Antioxidant activity of synthesized compounds.

The produced compounds’ antioxidant value was evaluated using a variety of tests, such as DPPH, and contrasted with that of butylated hydroxytoluene (BHT), which was used as a positive control and had a concentration of 50 µg/ml. Table 1 compound **15** exhibited significant DPPH activity with values of 44.44 ± 0.80, 16.50 ± 0.90, and 0.0 at concentrations of 2.0, 1.0, and 0.5 mg/mL, respectively. Additionally, compounds **4**, **11**, **12**, **13**, **14**, and **16** displayed increasing DPPH activity from 7.11 ± 0.2, 9.01 ± 0.88, 10.23 ± 1.03, 11.35 ± 0.98, 14.88 ± 1.00, and 8.21 ± 0.88 at 1.0 mg/mL to 26.52 ± 0.0, 28.34 ± 0.21, 30.64 ± 0.10, 35.62 ± 1.20, 36.52 ± 0.90, and 37.21 ± 1.80 at 2.0 mg/mL, respectively indicating its effectiveness in reducing oxidative stress at higher concentrations. Conversely, compounds **5**, **6**, **7**, **8**, **9**, and **10** demonstrated moderate antioxidant activities with DPPH values of 16.25 ± 0.10, 12.65 ± 0.21, 13.54 ± 0.21, 14.55 ± 0.13, 15.93 ± 0.33, and 16.87 ± 2.8 at 2.0 mg/ml. Compounds **1**, **2**, and **3** exhibited low DPPH activity at 2.0 mg/mL with scavenging activities of 8.10 ± 0.0, 10.23 ± 0.0, and 11.20 ± 0.08, respectively (Fig. 8). These substances have redox properties that contribute to their antioxidant qualities by enabling them to function as reducing agents, hydrogen atom contributors, and free radical scavengers. Either by electron donation or by partnering the DPPH odd electron with a hydrogen atom, these compounds exhibit antioxidant effects. Our findings align with Khalaf et al. (2025), who also evaluated the antioxidant activity of synthesized compounds using DPPH scavenging assays^48^. the antioxidant screening revealed a clear structure-activity trend, with compounds 13–16, particularly compound 15, exhibiting the most potent radical scavenging activity. At the test concentration of 2 mg/mL, compound 15 showed the highest activity, scavenging 44.44% of DPPH radicals. However, this activity is significantly lower than that of the standard antioxidant BHT. When compared at a standardized concentration for potency assessment, BHT at 50 µg/mL exhibited 68.22% scavenging activity. This highlights a substantial potency gap, as our lead compound 15 required a 40-fold higher mass concentration (2000 µg/mL) to achieve 44.44% scavenging. This comparison underscores that while the pyrimidoacetonitrile moiety in compound 15 confers notable antioxidant properties within this series, the absolute potency of these initial leads is moderate compared to a potent standard like BHT. Future work will focus on structural optimization to enhance this activity. The quantitative assessment of antioxidant activity, expressed as IC₅₀ values, established a clear and compelling structure-activity relationship (Table 1). The data identifies the pyrimidoacetonitrile moiety in compound 15 (IC₅₀ = 2.25 mg/mL) as the key structural feature conferring the most potent radical scavenging activity within this series.

**Table 1:** Antioxidant activity of synthesized compounds.

Compound	Antioxidant activity DPPH%			IC₅₀ mg ml^−1^
	2.0 mg ml^− 1^	1.0 mg ml^− 1^	0.5 mg ml^− 1^	
Control (BHT)	68.22 ± 2.8	44.62 ± 1.8	20.70 ± 1.8	1.46
**1**	8.10 ± 0.0	0.0	0.0	12.31
**2**	10.23 ± 0.0	0.0	0.0	9.77
**3**	11.20 ± 0.08	0.0	0.0	8.92
**4**	26.52 ± 0.0	7.11 ± 0.0	0.0	3.77
**5**	16.25 ± 0.10	0.0	0.0	6.15
**6**	12.65 ± 0.0.21	0.0	0.0	7.90
**7**	13.54 ± 0.0.21	0.00	0.0	7.34
**8**	14.55 ± 0.13	0.00	0.0	6.87
**9**	15.93 ± 0.33	0.00	0.0	6.22
**10**	16.87 ± 2.8	0.00	0.0	5.92
**11**	28.34 ± 0.21	9.01 ± 0.88	0.0	3.52
**12**	30.64 ± 0.10	10.23 ± 1.03	0.0	3.21
**13**	35.62 ± 1.20	11.35 ± 0.98	0.0	2.81
**14**	36.52 ± 0.90	14.88 ± 1.00	0.0	2.73
**15**	44.44 ± 0.80	16.50 ± 0.90	0.0	2.25
**16**	37.21 ± 0.1.80	8.21 ± 0.88	0.0	2.68

### Antibacterial activities of compounds towards pathogenic microbial strains

The antimicrobial (antibacterial and antifungal) properties of all produced compounds were tested against a variety of bacterial species (Table 2). When the antibacterial efficacy was evaluated against six different microorganisms, compounds 7, 9, 11, 12, and 15 showed activity against every bacterial strain that was tested, showcasing substantial inhibition zones with diameters of (10.8 ± 0.13, 8.0 ± 0.11, 9.5 ± 0.0, 6.5 ± 0.0 and 7.0 ± 0.05) for E.coli ATCC25915, (11.5 ± 0.05, 12.10 ± 0.00, 14.60 ± 0.00, 13.20 ± 0.00 and 10.50 ± 0.00 mm) for S. aureus ATCC25923, (8.5 ± 0.43, 9.1 ± 0.40, 6.5 ± 0.40, 10.3 ± 0.40 and 11.2 ± 0.30 mm) for E. faecalis ATCC29212, (12.5 ± 0.07, 2.0 ± 0.40, 14.0 ± 0.00, 8.60 ± 0.00, 10.0 ± 0.00, 12.1 ± 0.00 mm) for P. aeruginosa ATCC10145, and (16.5 ± 0.43, 10.01 ± 0.11, 12.01 ± 0.11, 8.00 ± 0.11, and 11.00 ± 0.11) mm for S. mutans ATCC 25,175 when compared to Ciprofloxacin. Additionally, Moreover, compounds **4** and **6** were active against three tested bacterial strain with moderate diameters of inhibition zones (2.3 ± 0.10 and 2.0 ± 0.90 mm) for S. aureus ATCC25923, (2.8 ± 0.11 and 2.0 ± 0.11 mm) for E. faecalis ATCC29212, (2.0 ± 0.14 and 2.5 ± 0.14 mm) for P. aeruginosa ATCC10145. On the other hand, compounds **3**, **10**, **13**, and **16** showed only activity against E. faecalis ATCC29212 with inhibition (2.5 ± 0.43, 3.0 ± 0.20, 3.0 ± 0.20, and 3.0 ± 0.20), respectively, Compound 14 also demonstrated moderate inhibitory zones (3.0 ± 0.23 mm) against S. aureus ATCC25923. Additionally, every chemical is not inhibited by the yeast strain Candida albicans (Table 2). In order to ascertain the minimum inhibitory concentration (MIC) values of compounds 7, 9, 11, 12, and 15 against pathogenic bacteria, the most active compounds were further assessed for their antibacterial properties, as shown in (Table 3). The MIC values for compound **15** were determined as 2.0, 1.0, 1.0, 1.0, and 1.0 mg/mL against E. coli ATCC25915, S. aureus ATCC25923, E. faecalis ATCC29212, P. aeruginosa ATCC10145, and S. mutans ATCC25175, respectively. Compound **11** and **12** exhibited MIC values of 2.0, 1.0, 2.0, 2.0, and 2.0 mg/mL against the same bacterial strains. When tested against E. coli ATCC25915, S. aureus ATCC25923, and P. aeruginosa ATCC10145, compounds 7 and 9 showed MIC values of 2.0, 1.0, and 2.0 mg/mL, respectively. Our data reveal that the MIC values for our lead compounds are in a highly relevant and potent range (250–1000 µg/mL), which allows for a more favorable comparison. Our lead compounds demonstrate compelling activity. For instance: Compound 11 and 12 show excellent potency against *S. aureus* (MIC = 250 µg/mL). While Compound 15 exhibits remarkable broad-spectrum potency, with MICs of 250 µg/mL against both *S. aureus* and *E. faecalis*, and 500 µg/mL against the other three strains, including the notoriously resistant *P. aeruginosa* (Table 4). MIC values in the 250–1000 µg/mL range are considered promising for novel antibacterial agents, especially for a first-reporting study. This level of potency provide a strong foundation for medicinal chemistry optimization to further improve potency and pharmacokinetics. Highly Relevant for Topical Application: For skin, wound, or burn infections, achieving high local concentrations is feasible. The MICs of our compounds are well within the range that makes them excellent candidates for development.

**Table 2:** Antibacterial and antifungal activities of compounds on yeast and bacterial strains.

Compounds (2 mg/mL)	Antimicrobial activities (mm)					
	C. albicans	E.coli ATCC25915	S. aureus ATCC25923	E. faecalis ATCC29212	*P*. aeruginosa ATCC10145	S. mutans ATCC 25,175
**1**	(–)	(–)	(–)	(–)	(–)	(–)
**2**	**(–)**	(–)	(–)	(–)	(–)	(–)
**3**	**(–)**	(–)	(–)	(+) 2.5 ± 0.43	(–)	(–)
**4**	**(–)**	(–)	(+) 2.3 ± 0.10	(+) 2.8 ± 0.11	(+) 2.0 ± 0.14	(–)
**5**	**(–)**	(–)	(–)	(–)	(–)	(–)
**6**	**(–)**	(–)	(+) 2.0 ± 0.90	(+) 2.0 ± 0.11	(+) 2.5 ± 0.14	(–)
**7**	**(–)**	(+) 10.8 ± 0.13	(+) 11.5 ± 0.05	(+) 8.5 ± 0.43	(+) 12.5 ± 0.07	(+) 16.5 ± 0.43
**8**	(–)	(–)	(+) 1.0 ± 0.10	(–)	(+) 2.0 ± 0.40	(–)
**9**	**(–)**	(+) 8.0 ± 0.11	(+) 12.10 ± 0.00	(+) 9.1 ± 0.40	(+) 14.0 ± 0.00	(+) 10.01 ± 0.11
**10**	(–)	(–)	(–)	(+) 3.0 ± 0.20	(–)	(–)
**11**	**(–)**	(+) 9.5 ± 0.0	(+) 14.60 ± 0.00	(+) 6.5 ± 0.40	(+) 8.60 ± 0.00	(+) 12.01 ± 0.11
**12**	**(–)**	(+) 6.5 ± 0.0	(+) 13.20 ± 0.00	(+) 10.3 ± 0.40	(+) 10.0 ± 0.00	(+) 8.00 ± 0.11
**13**	**(–)**	(–)	(–)	(+) 3.0 ± 0.20	(–)	(–)
**14**	**(–)**	(–)	(+) 3.0 ± 0.23	(–)	(–)	(–)
**15**	**(–)**	(+) 7.0 ± 0.05	(+) 10.50 ± 0.00	(+) 11.2 ± 0.30	(+) 12.1 ± 0.00	(+) 11.00 ± 0.11
**16**	**(–)**	(–)	(–)	(+) 3.0 ± 0.20	(–)	(–)
Ciprofloxacin (50 µg/mL)	**–**	(+) 12.00 ± 0.23	(+) 13.00 ± 0.11	(+) 12.45 ± 0.08	(+) 15.32 ± 0.54	(+) 15.66 ± 0.04
Fluconazole (100 µg/mL)	(+) 13.00 ± 0.11	–	–	–	–	–

Values are given as mean ± standard error.

**Table 3:** MIC of the most active compounds by agar diffusion methods.

Compounds	Concentration		Antibacterial activities (mm)			
		E. coli ATCC25915	S. aureus ATCC25923	E. faecalis ATCC29212	P. aeruginosa ATCC10145	S. mutans ATCC 25,175
**7**	2.0 mg/mL	(+) 10.8 ± 0.13	(+) 11.5 ± 0.05	(+) 8.5 ± 0.43	(+) 12.5 ± 0.07	(+) 16.5 ± 0.43
	1.0 mg/mL	(–)	(+) 4.5 ± 0.00	(–)	(+) 5.20 ± 0.01	(+) 5.0 ± 0.03
	0.5 mg/mL	(–)	(–)	(–)	(–)	(–)
**9**	2.0 mg/mL	(+) 8.0 ± 0.11	(+) 12.10 ± 0.00	(+) 9.1 ± 0.40	(+) 14.0 ± 0.00	(+) 10.01 ± 0.11
	1.0 mg/mL	(–)	(+) 6.00 ± 0.05	(–)	(+) 7.10 ± 0.05	(–)
	0.5 mg/mL	(–)	(–)	(–)	(–)	(–)
**11**	2.0 mg/mL	(+) 9.5 ± 0.0	(+) 14.60 ± 0.00	(+) 6.5 ± 0.40	(+) 10.0 ± 0.00	(+) 12.01 ± 0.11
	1.0 mg/mL	(–)	(+) 8.0 ± 0.15	(–)	(–)	(–)
	0.5 mg/mL	(–)	(–)	(–)	(–)	(–)
**12**	2.0 mg/mL	(+) 6.5 ± 0.0	(+) 13.20 ± 0.00	(+) 10.3 ± 0.40	(+) 10.0 ± 0.00	(+) 8.00 ± 0.11
	1.0 mg/mL	(–)	(+) 5.10 ± 0.00	(–)	(–)	(–)
	0.5 mg/mL	(–)	(–)	(–)	(–)	(–)
**15**	2.0 mg/mL	(+) 7.0 ± 0.05	(+) 10.50 ± 0.00	(+) 11.2 ± 0.30	(+) 12.1 ± 0.00	(+) 11.00 ± 0.11
	1.0 mg/mL		(+) 3.50 ± 0.00	(+) 4.80 ± 0.00	(+) 6.70 ± 0.00	(+) 4.50 ± 0.00
	0.5 mg/mL	(–)	(–)	(–)	(–)	(–)

**Table 4:** MIC of the most active compounds by broth dilution.

Compounds	MIC of the most active compounds (µg/mL)				
	E. coli ATCC25915	S. aureus ATCC25923	E. faecalis ATCC29212	*P*. aeruginosa ATCC10145	S. mutans ATCC 25,175
**7**	500	500	1000	500	500
**9**	1000	500	1000	500	1000
**11**	1000	250	1000	1000	1000
**12**	1000	250	1000	500	1000
**15**	500	250	250	500	500

Values are given as mean ± standard error.

### Docking and molecular interaction research using S. aureus’s dihydropteroate synthase

**Fig. 9:**
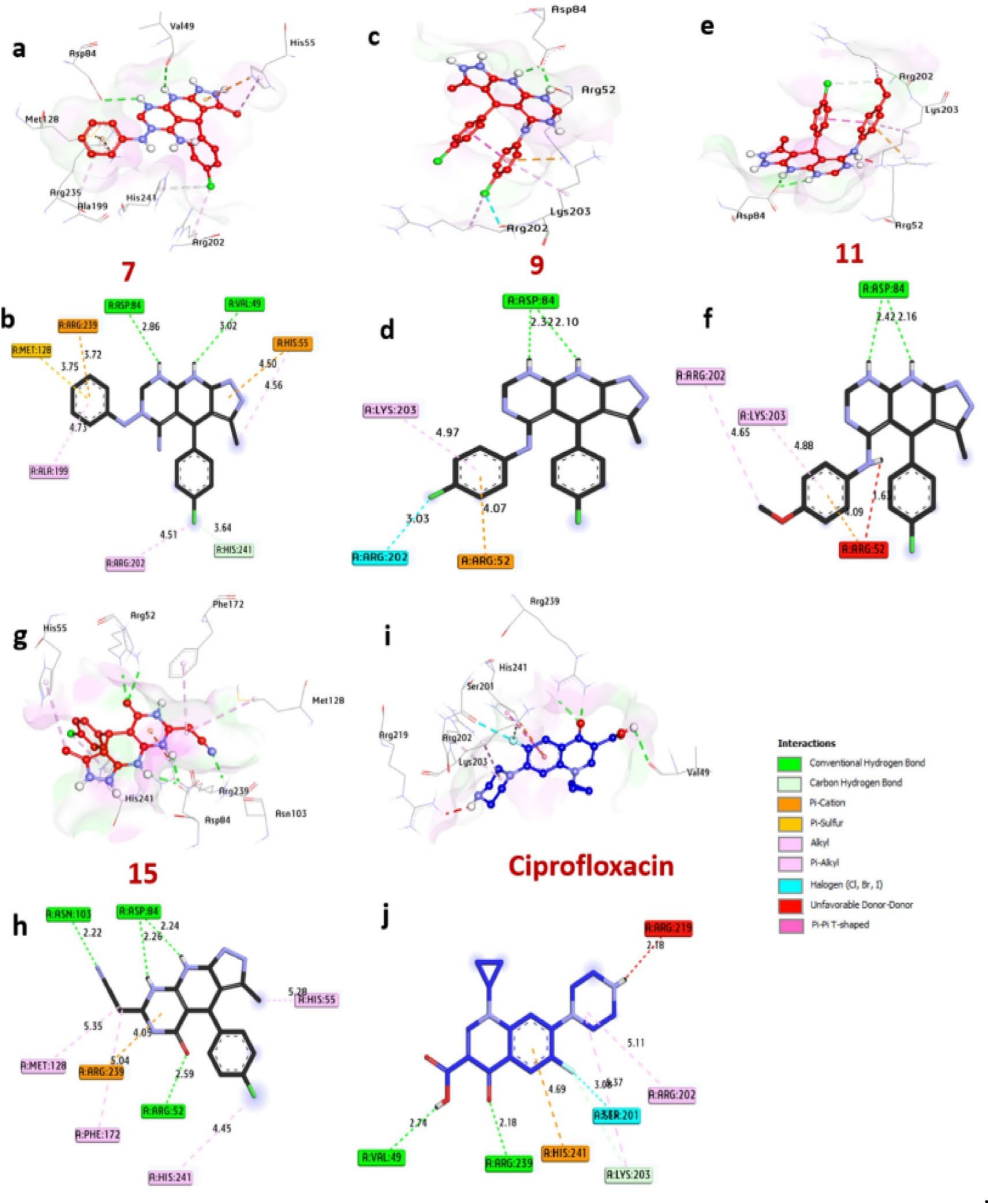
Images of the molecule in three dimensions at the binding pocket of S. aureus dihydropteroate synthase (PDB: ID 1AD4). These are (**a** and **b**) 7, (**c** and **d**) 9, (**e** and **f**) 11, (**g** and **h**) 15, and (**i** and **j**) ciprofloxacin.

The dihydropteroate synthase of S. aureus, an essential enzyme in the folate production pathway required for bacterial growth, was used to measure the binding energies of the produced compounds. Figure 9 shows two– dimensional representations of the compounds.

In contrast to ciprofloxacin (-6.10 kcal/mol), compounds 7, 9, 11, and 15 showed favorable binding energies of -7.80, -7.70, -7.70, and − 8.50 kcal/mol, respectively. These substances were discovered to establish hydrogen bonds with a number of amino acids, including Arg52, Asp84, Val49, and Asn103. They also conducted hydrophobic interactions in the activity pocket, such as alkyl bonds with His55, Ala199, His241, Lys203, Arg202, Phe172, and Met128; pi-sulfur interactions with Met128; CH-bond interactions with His241; pi-cation interactions with Arg239; and halogen interactions with Arg202. Furthermore, it was shown that the catalytic site’s amino acids, such as Arg52, His55, and Val49, increased the compounds’ binding affinities. Therefore, it is probable that compounds 7, 9, 11, and 15 will have their antibacterial action by successfully blocking the dihydropteroate synthase enzyme in S. aureus (Table 5).

**Table 5:** Ligand-amino acid interactions with S. aureus dihydropteroate synthase (PDB: ID 1AD4).

	Protein	Ligand	Hydrophilic interactions	Hydrophobic contacts			No. of H-bonds	No. of total bonds	Affinity kcal mol^-1^
			Residue (H-bond)	Length	Residue (bond type)	Length			
1	Dihydropteroate synthase of S. aureus	**7**	Val49 (H-Bond) Asp84 (H-Bond)	3.02 2.86	His55, (Pi-alkyl)	4.51	2	9	− 7.80
					His55, (Pi-cation)	4.50			
					Arg239, (Pi-cation)	3.72			
					Ala199, (Pi-alkyl)	4.73			
					Arg202, (Pi-alkyl)	4.51			
					Met128, (Pi-Sulfur)	3.75			
					His241, (CH-bond)	3.64			
2		**9**	Asp84, (H-Bond) Asp84, (H-Bond)	2.10 2.32	Lys203, (Pi-alkyl)	4.97	2	5	− 7.70
					Arg239, (Pi-cation)	4.07			
					Arg202, (Halogen)	3.03			
3		**11**	Asp84, (H-Bond) Asp84, (H-Bond)	2.16 2.42	Lys203, (Pi-alkyl)	4.88	2	6	− 7.70
					Arg202, (Pi-alkyl)	4.65			
					Arg239, (Pi-cation)	4.09			
4		**15**	Asp84 (H-Bond) Asp84 (H-Bond) Asn103 (H-Bond) Arg52 (H-Bond)	2.26 2.26 2.22 2.59	His55, (Pi-alkyl)	5.28	4	9	− 8.50
					His241, (Pi-alkyl)	4.45			
					Arg239, (Pi-cation)	4.09			
					Phe172, (Pi-alkyl)	5.04			
					Met128, (Pi-alkyl)	5.35			
5		**Ciprofloxacin**	Val49, (H-Bond) Arg239 (H-Bond)	2.74 2.18	Ser201, (Halogen)	3.02	2	8	− 6.10
					Arg202, (Pi-alkyl)	5.11			
					His241, (Pi-cation)	4.69			
					Lys203, (Pi-alkyl)	5.37			
					Lys203, (CH-bond)	3.55			
					Arg219, (Unfavorable)	2.18			

### Studies of the LasR protein’s docking and molecular interactions in P. aeruginosa

**Fig. 10:**
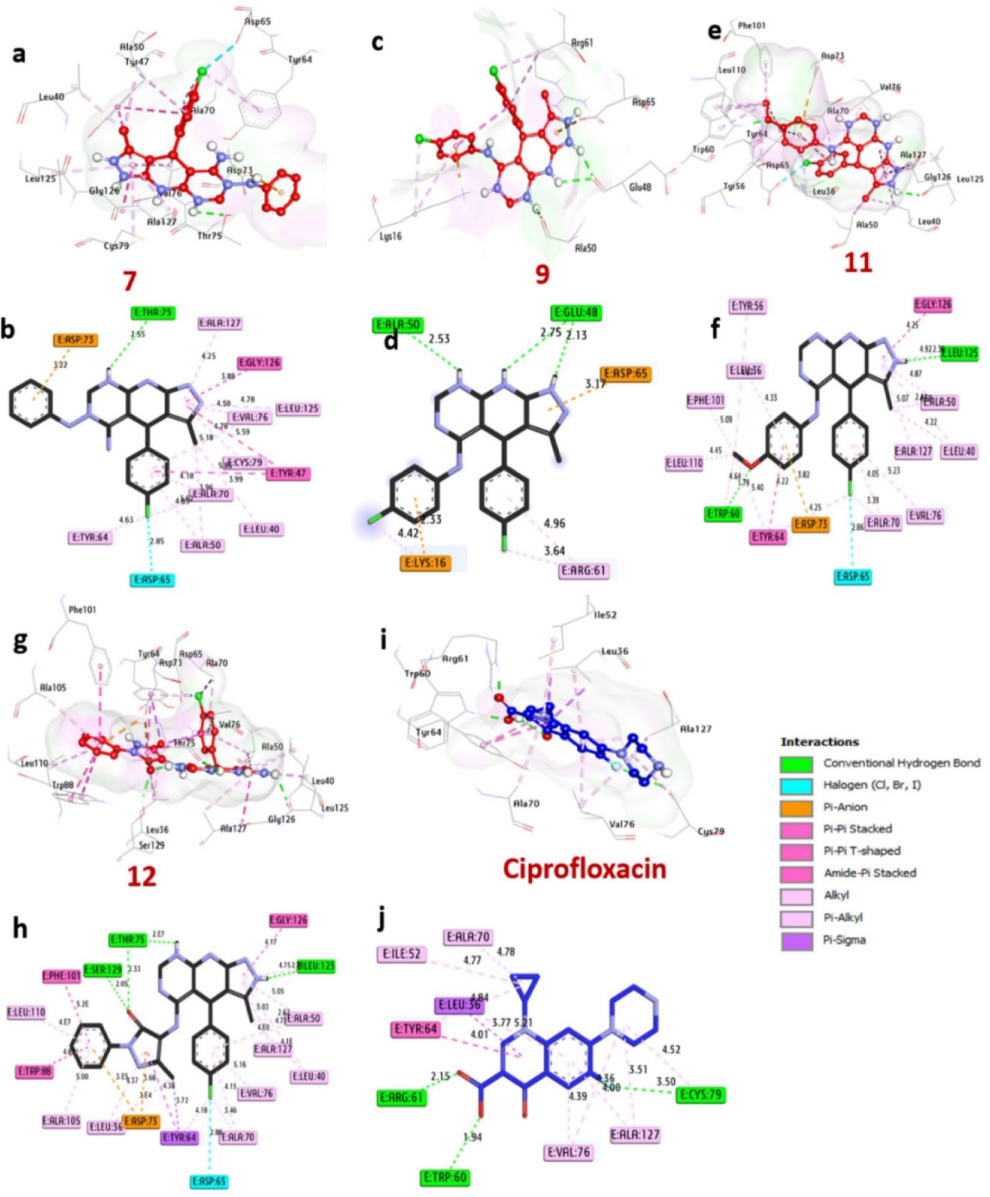
Compounds in the LasR protein binding pocket in P. aeruginosa (PDB: ID 2UV0) are shown in three dimensions as follows: (**a** and **b**) 7, (**c** and **d**) 9, (**e** and **f**) 11, (**g** and **h**) 12, and (**i** and **j**) ciprofloxacin.

Table 6 summarizes and (Fig. 10) illustrates the docking results of specific compounds and ciprofloxacin against the LasR protein, a transcriptional regulator for pathogenicity in P. aeruginosa. Among the compounds tested, the most potent bacterial inhibitors, compounds 7, 9, 11, and 12, exhibited significant affinity interactions with binding energies of -9.50, -8.50, -9.50, and − 10.20 kcal/mol, respectively, compared to ciprofloxacin (-8.50 kcal/mol). It was discovered that these substances formed hydrogen bonds with important amino acids, including Ser129, Glu48, Trp60, Leu125, Thr75, and Ala50. Additionally, they formed alkyl bonds with Ala127, Val76, Leu125, Leu40, Ala70, Cys79, Ala50, Tyr64, and other hydrophobic contacts inside the activity pocket. Pi-anion with Asp73, halogen bonds with Asp65, pi-cation contacts with Asp65 and Lys16, pi-pi T-shaped interactions with Gly126, Tyr47, Trp88, Phe101, and other elements, and pi-sigma interactions with Tyr64 were among the other interactions. Moreover, the binding affinity of these compounds was found to be increased by amino acids found in the catalytic region, such as Asp73, Gly77, Thr165, Thr75, Glu48, and Ser129. The validated in vitro antibacterial activity results and these molecular docking studies indicate that compounds 7, 9, 11, and 12 may be effective bacterial inhibitors that target the LasR protein in P. aeruginosa.

**Table 6:** Ligand-LasR protein molecular interactions in P. aeruginosa.

Protein	Ligand	Hydrophilic interactions	Hydrophobic contacts			No. of H-bonds	No. of total bonds	Affinity kcal mol^−1^
		Residue (H-Bond)	Length	Residue (bond type)	Length			
LasR protein in P. aeruginosa	**7**	Thr75, (H-Bond)	2.55	Asp73, (Pi-Anion)	3.22	1	13	− 9.50
				Asp65, (Halogen)	2.85			
				Gly126, (Pi-Pi T shaped)	3.89			
				Tyr47, (Pi-Pi T shaped)	5.86			
				Ala127, (Pi-alkyl)	4.25			
				Val76, (Pi-alkyl)	4.50			
				Leu125, (Pi-alkyl)	4.78			
				Leu40, (Pi-alkyl)	3.99			
				Ala70, (Pi-alkyl)	3.96			
				Cys79, (Pi-alkyl)	4.78			
				Ala50, (Pi-alkyl)	4.88			
				Tyr64, (Pi-alkyl)	4.63			
	**9**	Ala50, (H-Bond) Glu48, (H-Bond) Glu48, (H-Bond)	2.53 2.75 2.13	Asp65, (Pi-cation)	3.17	3	8	− 8.50
				Lys16, (Pi-alkyl)	4.42			
				Arg61, (Pi-alkyl)	3.64			
				Arg61, (Pi-alkyl)	4.96			
				Lys16, (Pi-Anion)	2.33			
	**11**	Leu125, (H-Bond) Trp60, (H-Bond)	2.33 1.79	Asp73, (Pi-Anion)	3.82	2	21	− 9.5
				Asp65, (Halogen)	2.86			
				Gly126, (Pi-Pi T shaped)	4.25			
				Tyr47, (Pi-Pi T shaped)	4.22			
				Ala127, (Pi-alkyl)	5.07			
				Val76, (Pi-alkyl)	5.23			
				Leu110, (Pi-alkyl)	4.45			
				Leu40, (Pi-alkyl)	4.22			
				Ala70, (Pi-alkyl)	4.05			
				Ala50, (Pi-alkyl)	4.87			
				Tyr64, (Pi-alkyl)	5.09			
				Phe101, (Pi-alkyl)	5.09			
				Leu36, (Pi-alkyl)	4.33			
	**12**	Leu125, (H-Bond) Thr75, (H-Bond) Thr75, (H-Bond) Ser129, (H-Bond)	5.20 2.77 2.33 2.05	Asp73, (Pi-Anion)	3.54	4	20	− 10.20
				Asp65, (Halogen)	2.86			
				Gly126, (Pi-Pi T shaped)	4.17			
				Trp88, (Pi-Pi T shaped)	4.88			
				Phe101, (Pi-Pi T shaped)	5.20			
				Tyr64, (Pi-sigma)	3.72			
				Ala127, (Pi-alkyl)	4.30			
				Val76, (Pi-alkyl)	5.16			
				Leu110, (Pi-alkyl)	4.07			
				Leu40, (Pi-alkyl)	4.10			
				Ala70, (Pi-alkyl)	3.46			
				Ala50, (Pi-alkyl)	5.05			
				Tyr64, (Pi-alkyl)	4.18			
				Ala105, (Pi-alkyl)	5.00			
				Leu36, (Pi-alkyl)	3.44			
	**Ciprofloxacin**	Cys79, (H-Bond) Arg61, (H-Bond) Trp60, (H-Bond)	3.50 2.15 1.94	Ala70, (Pi-alkyl)	4.78	3	12	− 8.50
				Ile52, (Pi-alkyl)	4.77			
				Val76, (Pi-alkyl)	4.39			
				Ala127, (Pi-alkyl)	4.00			
				Tyr64, (Pi-Pi T shaped)	4.01			
				Leu36, (Pi-sigma)	3.77			

### Docking and molecular interaction research using E. coli DNA gyrase

**Fig. 11:**
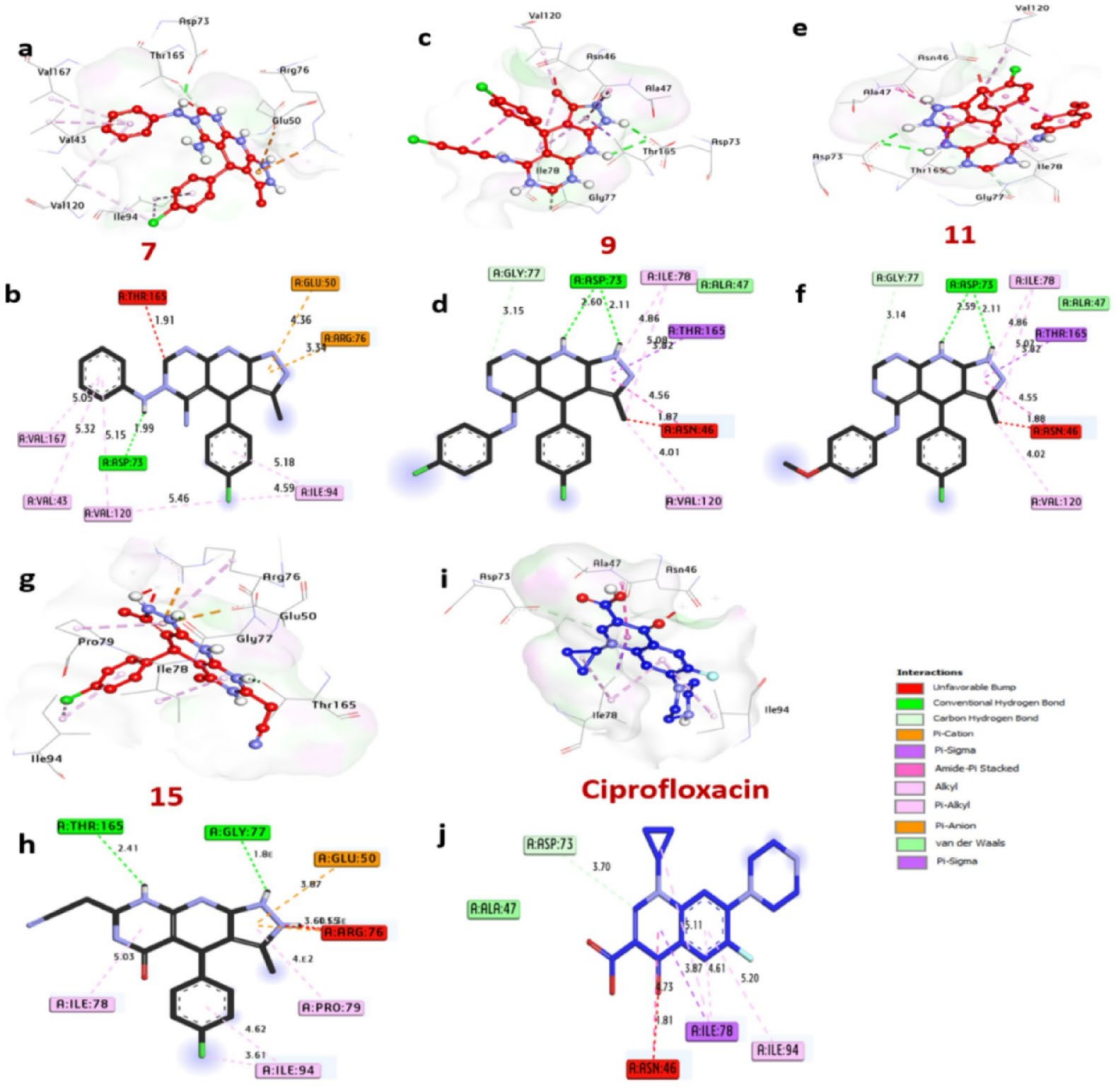
Compounds at the binding pocket of E. coli DNA gyrase (PDB: ID 7P2M) are shown in three dimensions: (**a** and **b**) 7, (**c** and **d**) 9, (**e** and **f**) 11, (**g** and **h**) 15, and (**i** and **j**) ciprofloxacin.

As shown in (Table 7) and (Fig. 11), the interactions of the DNA gyrase enzyme, which is essential for transcription and DNA replication in bacteria such as E. coli, with certain compounds and the reference antibiotic ciprofloxacin were examined. The docking results are presented in (Table 7) and (Fig. 11), highlighting the best bacteria inhibitors: compounds **7**, **9**, **11**, and **15**, with notable affinity interactions of -8.90, -8.0, -7.7, and − 8.0 kcal/mol, respectively, compared to -6.6 kcal/mol for Ciprofloxacin. It was noted that these substances formed hydrogen bonds with important amino acids like Thr165, Gly77, and Asp73. In the activity pocket, they also formed a variety of hydrophobic interactions, such as alkyl bonds with Ile94, Val120, Val43, Val167, Ile78, and Pro79, Pi-Pi T shaped interactions with Asn46, Pi-Sigma interactions with Thr165, C-H bond interactions with Gly77, and Pi-cation interactions with Glu50 and Arg76Moreover, it was discovered that the catalytic site’s amino acids, such as Asp73, Gly77, and Thr165, increased the compounds’ binding affinities. By focusing on the DNA gyrase enzyme, chemicals 7, 9, 11, and 15 may be effective bacterial growth inhibitors, according to the docking analysis’s overall results.

**Table 7:** Ligand-amino acid interactions with DNA gyrase of E. coli (PDB: ID 7P2M).

No	Protein	Ligand	Hydrophilic interactions		Hydrophobic contacts		No. of H-bonds	No. of total bonds	Affinity kcal mol^−1^
			Residue (H-bond)	Length	Residue (bond type)	Length			
1	DNA Gyrase of E.coli (PDB: ID 7P2M)	**7**	Asp73, (H- Bond)	1.99	Ile94, (alkyl)	5.18	**1**	**10**	**− 8.90**
					Ile94, (alkyl)	4.95			
					Val120, (alkyl)	5.46			
					Thr165, (Unfavorable)	1.91			
					Val43, (alkyl)	5.32			
					Val167, (alkyl)	5.05			
					Val120, (alkyl)	5.15			
					Glu50, ((pi-Cation)	4.36			
					Arg76, ((pi-Cation)	3.34			
2		**9**	Asp73, (H- Bond) Asp73, (H- Bond)	2.11 2.60	Val120, (alkyl)	4.01	**2**	**8**	**− 8.0**
					Ile78, (alkyl)	4.86			
					Ile78, (alkyl	5.08			
					Asn46, (Pi-Pi T shaped)	4.56			
					Thr165, (pi-Sigma)	3.82			
					Gly77, (C-H bond)	3.15			
		**11**	Asp73, (H- Bond) Asp73, (H- Bond)	2.11 2.59	Val120, (alkyl)	4.02	**2**	**8**	**− 7.7**
					Ile78, (alkyl)	4.86			
					Ile78, (alkyl	5.07			
					Asn46, (Pi-Pi T shaped)	4.55			
					Thr165, (pi-Sigma)	3.82			
					Gly77, (C-H bond)	3.14			
		**15**	Gly77, (H- Bond) Thr165, (H- Bond)	1.80 2.41	Ile78, (alkyl)	5.03	**2**	**8**	**− 8.0**
					Ile94, (alkyl)	3.61			
					Ile94, (alkyl	4.62			
					Pro79, (alkyl)	4.02			
					Glu50, (pi-Cation)	3.87			
					Arg76, (pi-Cation)	3.60			
3		**Ciprofloxacin**	**–**	–	Asn46, (Unfavorable)	1.81	**0**	**7**	**− 6.6**
					Ile94, (alkyl)	5.20			
					Ile78, (alkyl)	4.61			
					Ile78, (alkyl	3.87			
					Asn46, (Pi-Pi T shaped)	4.73			
					Ile78, (pi-Sigma)	3.87			
					Asp73, (C-H bond)	3.70			

### Docking and interaction investigations of E. faecalis’s KPC-2 carbapenemase

**Fig. 12:**
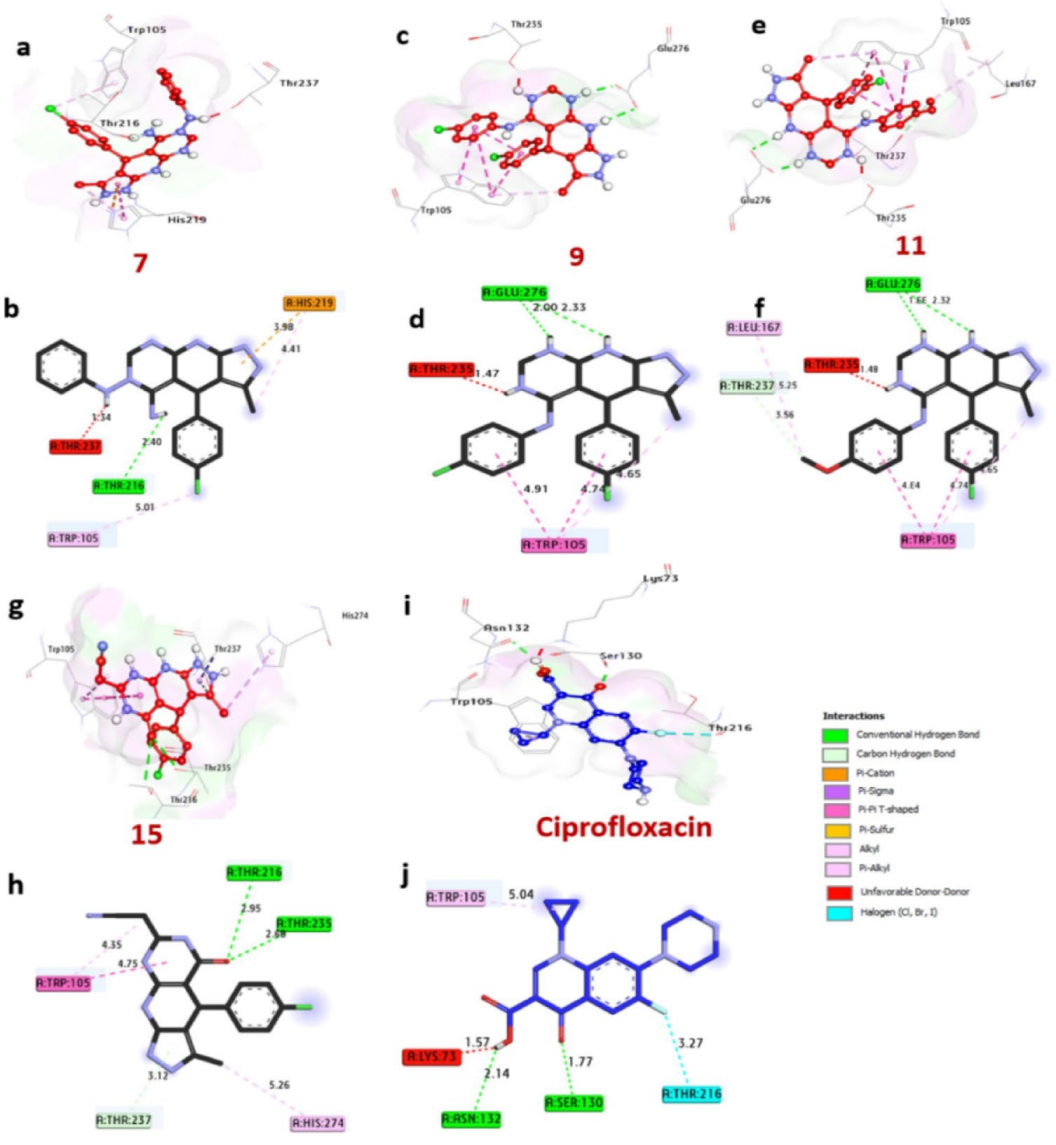
3D representations of compounds at the binding pocket of KPC-2 carbapenemase of E. faecalis (PDB: ID 2OV5): (**a** and** b**) **7**, (**c** and** d**) **9**, (**e** and** f**) **11**, (**g** and** h**) **12**, and (**i** and** j**) ciprofloxacin.

In the fight against bacteria that are resistant to several drugs, E. faecalis’s KPC-2 carbapenemase enzyme plays a vital role in providing resistance to carbapenem antibiotics. (Table 8) provides specifics on the docking results of a few chosen compounds with the reference antibiotic ciprofloxacin, whereas Fig. 12 shows the results. When compared to the control gentamicin (-5.3 kcal/mol), the most potent bacteria inhibitors among the compounds examined, compounds 7, 9, 11, and 15, showed significant affinity interactions of -5.9 and − 5.7 kcal/mol, respectively. These substances were discovered to establish hydrogen bonds with important amino acids, including Glu276, Thr216, and Thr235. Additionally, they engaged in a number of hydrophobic interactions inside the activity pocket, including C-H bond interactions with Thr237, Pi-cation contacts with His219, Pi-Pi T-shaped interactions with Trp105, and alkyl bonds with Trp105, His219, His274, and Leu167. Additionally, it was found that the catalytic site’s amino acids, such as Glu276 and Thr216 and Thr235, increased the compounds’ binding affinities. Compounds 7, 9, 11, and 15 appear to be promising as strong bacterial inhibitors that target the KPC-2 enzyme in E. faecalis, according to the combined data from the docking studies and verified in vitro antibacterial activity results.

**Table 8:** Ligand-amino acid interactions with KPC-2 carbapenemase of K. pneumonia (PDB: ID 2OV5).

No	Protein	Ligand	Hydrophilic Interactions		Hydrophobic contacts		No. of H-Bonds	No. of total bonds	Affinity kcal mol-1
			Residue (H-Bond)	Length	Residue (bond type)	Length			
1	KPC-2 carbapenemase of K. pneumoniae (PDB: ID 2OV5)	**7**	Thr216, (H-Bond)	2.81	Trp105, (Pi-alkyl) His219, (Pi-alkyl) His219, (Pi-Cation)	5.01 4.41 3.98	1	4	**− 5.9**
2		**9**	Glu276, (H-Bond) Glu276, (H-Bond)	2.33 2.00	Trp105, (Pi-Pi T shaped) Trp105, (Pi-Pi T shaped) Trp105, (alkyl)	4.74 4.91 4.65	2	5	**− 5.7**
		**11**	Glu276, (H-Bond) Glu276, (H-Bond)	2.32 1.77	Trp105, (Pi-Pi T shaped) Trp105, (Pi-Pi T shaped) Trp105, (alkyl) Leu167, (alkyl) Thr237, (C-H bond)	4.74 4.74 4.65 5.25 3.56	2	7	**− 5.7**
		**15**	Thr216, (H-Bond) Thr235, (H-Bond)	2.95 2.68	Trp105, (Pi-Pi T shaped) Trp105, (Pi-alkyl) His274, (alkyl) Thr237, (C-H bond)	4.75 4.35 5.26 3.12	2	6	**− 5.7**
6		**Ciprofloxacin**	Ser130, (H-Bond) Asn132, (H-Bond)	1.77 2.14	Thr216, (Halogen) Trp105, (Pi-alkyl)	3.27 5.04	2	4	**− 5.3**

### In silico ADME prediction of synthesized compounds

**Fig. 13:**
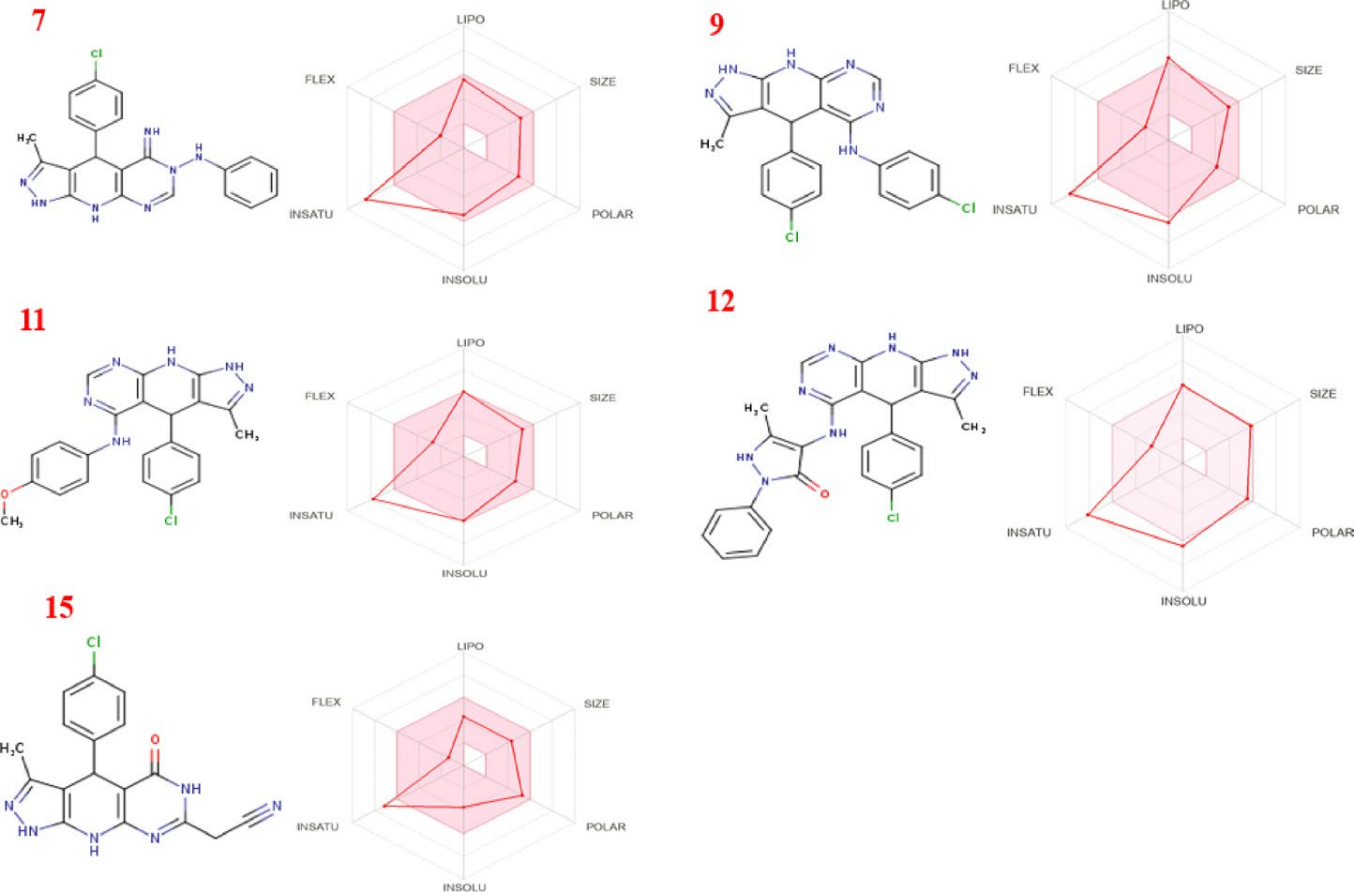
Oral bio-availability graph for compounds **7**,** 9**,** 11**,** 12**, and **15** produced with the help of the Swiss ADME tool.

**Fig. 14:**
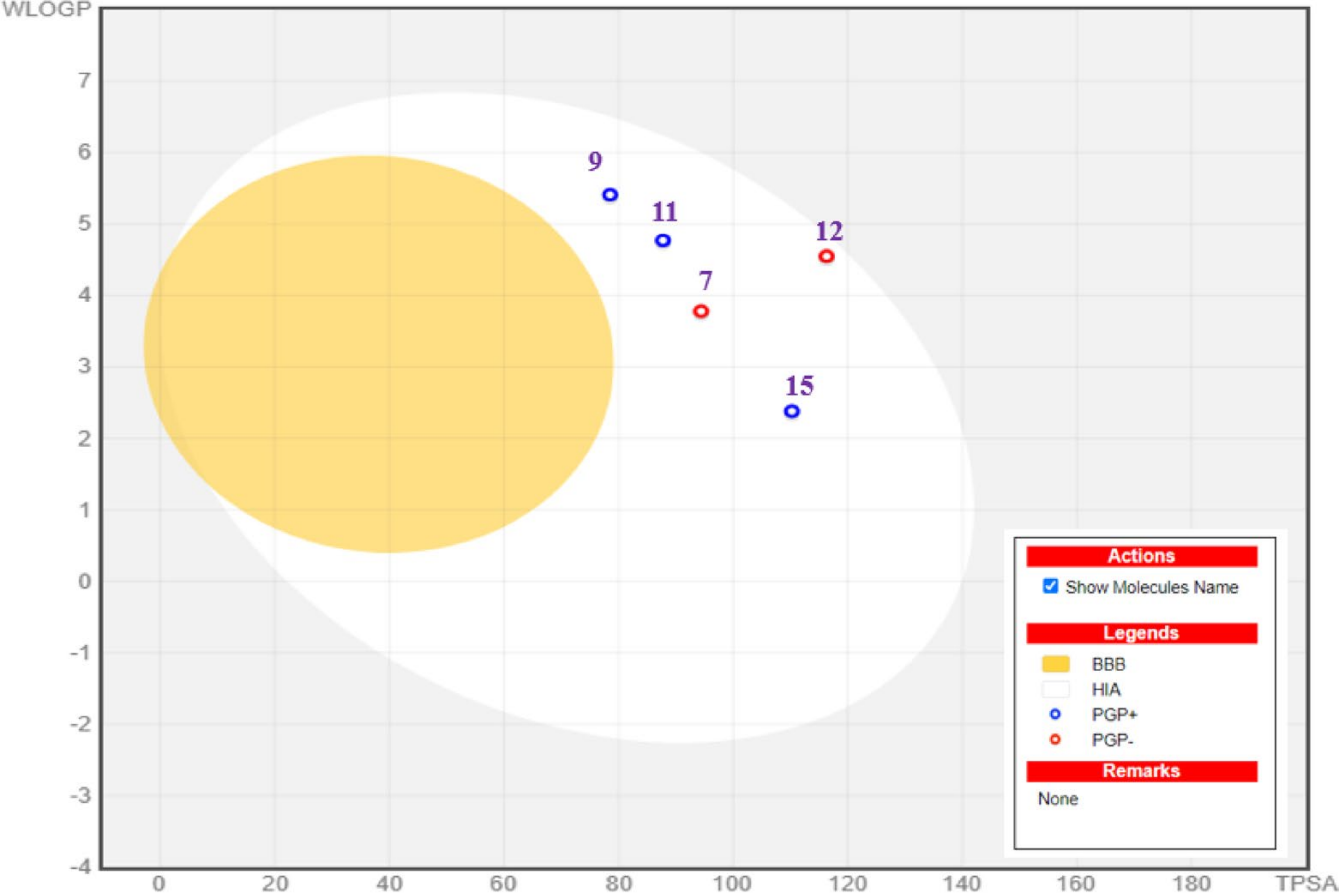
The boiled egg model for **7**, **9**, **11**, **12**, and **15**.

The promising qualities of the compounds in our investigation are highlighted by the physiochemical and ADME analyses presented in (Table 9) and (Fig. 13). With molecular weights of about 500, these compounds exhibit Lipinski compatibility, indicating their tiny size, ease of transferability, and effective absorption. Interestingly, they have a balanced ratio of hydrogen bond acceptors (HBA) and donors (HBD) (< 5 HBA, < 3 HBD), which increases the possibility of oral bioavailability, as well as a</mark > favorable number of rotatable bonds (2–5) that are essential for structural flexibility. Regarding water solubility and lipophilicity, the majority of substances have limited water solubility, except for compound 15, whose solubility is good. The allowed range for the lipophilicity parameter XLOGP3 is 2.03 to 5.69. The combination of high predicted GI absorption, compliance with key drug-likeness rules, and acceptable TPSA values suggests favorable prospects for oral bioavailability, though this must be confirmed experimentally with models like PAMPA or Caco-2 assays. compounds are not crossing the blood-brain barrier. All lead compounds (7, 9, 11, 12, 15) are predicted to have high gastrointestinal (GI) absorption, which is a positive indicator for potential oral bioavailability. Moreover, they show promise in inhibiting CYP2C9 and CYP3A4 enzymes, as depicted in (Fig. 14). Several compounds meet the strict requirements through thorough drug-likeness assessments using a variety of criteria, such as Lipinski, Ghose, Veber, Muegge, and Egan guidelines. This highlights their attractive physicochemical characteristics for possible drug development. Although the majority of chemicals show signs of irritancy, mutagenicity, tumorigenicity, or reproductive toxicity, their topological polar surface area (TPSA) values fall within the ideal range of 78.52–110.8, which suggests that they have good oral bioavailability and gastrointestinal absorption. Comparing compound 12 to its equivalents, it is noteworthy that it has higher drug ratings, indicating promising potential as an antibacterial drug-like agent. All things considered, these results point to the compounds’ great potential as medication candidates with high theoretical bioavailability, especially in the field of antibacterial therapies (Table 10). The in silico ADMET profile paints a promising yet nuanced picture of these antibacterial candidates. They possess excellent characteristics for oral bioavailability and a safety profile devoid of major red flags for genotoxicity. The predicted lack of BBB penetration is advantageous for minimizing neurotoxicity. The most significant identified risk is the potential for CYP-mediated drug-drug interactions with specific compounds (9, 11), which provides a clear directive for future lead optimization efforts. Compound 7, in particular, emerges as a standout lead due to its potent antibacterial activity, favorable binding mode in docking studies, and a cleaner CYP inhibition profile. Overall, these predictions strongly support the continued investigation of these compounds, with a focused strategy on mitigating the specific pharmacokinetic risks identified.

**Table 9:** Prediction of pharmacokinetics and physicochemical properties of compounds.

	ID	7	9	11	12	15
Physicochemical properties	MW	403.87	423.3	418.88	484.94	352.78
	Atoms	29	29	30	35	25
	Heavy atoms	23	23	23	28	17
	Csp3	0.1	0.1	0.14	0.12	0.18
	Rotatable bonds	3	3	4	4	2
	H-BA	3	3	4	4	4
	H-BD	4	3	3	4	3
	Molar refractivity	116.13	118.68	120.17	138.59	96.48
	TPSA	94.41	78.52	87.75	116.31	110.25
Lipophilicity and water solubility	iLOGP	2.16	2.58	2.52	2.48	1.04
	XLOGP3	4.3	5.69	5.03	5.2	2.03
	WLOGP	3.78	5.41	4.77	4.55	2.38
	MLOGP	4.24	4.38	3.58	4.02	2.14
	Silicos-IT Log P	3.29	4.7	4.11	4.15	3.87
	C. Log P	3.55	4.55	4	4.08	2.29
	ESOL Log S	− 5.44	− 6.44	− 5.91	− 6.45	− 3.68
	Ali Log S	− 6	− 7.1	− 6.61	− 7.39	− 3.97
	Silicos-IT class	Moderately	Poorly	Poorly	Poorly	Soluble
Pharmacokinetics	GI absorption	High	High	High	High	High
	BBB permeant	No	No	No	No	No
	Pgp substrate	No	Yes	Yes	No	Yes
	CYP1A2 inhibitor	No	Yes	No	Yes	Yes
	CYP2C19 inhibitor	Yes	Yes	Yes	Yes	Yes
	CYP2C9 inhibitor	No	No	Yes	No	No
	CYP2D6 inhibitor	No	Yes	Yes	No	No
	CYP3A4 inhibitor	No	Yes	Yes	No	No
	Skin permeation	− 5.71	− 4.84	− 5.28	− 5.57	− 7.01
Drug likeness	Lipinski	1	1	0	0	0
	Ghose	0	0	0	2	0
	Veber	0	0	0	0	0
	Egan	0	0	0	0	0
	Muegge	0	1	1	1	0
	Bioavailability	0.55	0.55	0.55	0.55	0.55
	Lead likeness	2	2	2	2	1

**Table 10:** Prediction of toxicity risks and physicochemical properties of compounds 7, 9, 11, 12, and 15.

o	Ligand	Toxicity risks				Physicochemical properties					
		Mutagenic	Tumorigenic	Irritant	Reproductive	CLogP	Solubility	Molecular Weight	TPSA	Drug likeness	Drug score
1	**7**	(–)	(–)	(–)	(–)	2.48	− 5.10	403.0	92.19	4.60	0.37
2	**9**	(–)	(–)	(–)	(–)	4.88	− 6.91	422.0	78.52	3.39	0.36
3	**11**	(–)	(–)	(–)	(–)	4.20	− 6.2	418.0	87.75	1.73	0.42
4	**12**	(–)	(–)	(–)	(–)	2.81	− 6.41	484.0	110.8	5.64	0.44
5	**15**	(–)	(–)	(–)	(–)	0.84	− 5.56	352.0	105.9	− 0.48	0.43

### Relationship between structure and activity (SAR)

The type of aromatic rings added to the core moieties had a significant impact on the antibacterial and antioxidant properties, according to the association between the identified activities of the examined compounds and their particular structural makeup. From the structure–activity relationship (SAR) view, compounds **7**, **9**, **11**, and **12**, containing N-substitutes phenyl rings, exhibited strong antimicrobial activity against various bacterial strains. This indicates that the presence of the phenyl ring substitutions at the nitrogen position enhances the compounds’ interaction with bacterial targets, thus increasing their antimicrobial efficacy. Compound **15** containing a pyrimidoacetonitrile moiety, exhibited notable DPPH radical scavenging activity, highlighting its strong antioxidant potential. This suggests that the pyrimidoacetonitrile group plays a crucial role in neutralizing free radicals, which could make this compound a promising candidate for further development as an antioxidant agent. The SAR explicitly uses MIC values (µg/mL) and binding energies (kcal/mol) to draw clear, quantitative correlations between structure and activity. Compound 11, featuring a p-chlorophenyl group, displayed the most potent activity against *S. aureus* with an MIC of 250 µg/mL and the most favorable binding energy (-7.70 kcal/mol) against dihydropteroate synthase, outperforming the other compounds in this series. Similarly, Compound 15, with its unique pyrimidoacetonitrile moiety, showed a balanced and potent MIC profile (250–500 µg/mL across all strains) and the strongest binding affinity (-8.50 kcal/mol) for the same enzyme, suggesting a highly efficient target engagement.

- Antimicrobial efficacy is highly dependent on the nature of the substituents attached to the core scaffolds, with dramatic differences observed between different substitution patterns.
- The Critical Importance of an N-Substituted Phenyl Ring the most significant finding is that the most potent broad-spectrum antibacterial activity is conferred by an N-substituted phenyl ring on the pyrazolopyridopyrimidine core. This is evident when comparing the highly active compounds 7, 9, 11, and 12 to their inactive or weakly active counterparts. Compounds 1, 2, 5, 8, 10, 13, 14, and 16, which lack this specific substitution, show no or negligible activity against most bacterial strains. The N-phenyl group provides optimal hydrophobicity and planar geometry for penetrating bacterial membranes and interacting with target enzymes, dihydropteroate synthase and DNA gyrase.
- Influence of the second aromatic moiety: within the series of N-phenyl substituted compounds (7, 9, 11, 12), the nature of the second aromatic ring fine-tunes the potency and spectrum. Phenyl vs. Pyridyl: Compound 7 exhibits robust, balanced activity across all bacterial strains. Replacing this phenyl with a pyridyl ring in compound 9 results in a similar broad-spectrum profile but with slightly varied potency (enhanced activity against *P. aeruginosa* but reduced activity against *S. mutans*). This suggests that the hydrogen-bond accepting potential of the pyridine nitrogen can modulate interactions with different bacterial targets. The most potent antibacterial activity, particularly against Gram-positive bacteria, is observed when the second aromatic ring contains strong electron-withdrawing groups. Compound 11 (with a p-chlorophenyl group) and compound 12 (with a p-bromophenyl group) show the strongest activity against *S. aureus* (14.60 and 13.20 mm, respectively). Halogen atoms (Cl, Br) enhance target binding through halogen bonding and by increasing lipophilicity, improving membrane penetration.
- The Unique case of the pyrimidoacetonitrile Moiety: Compound 15, which features a pyrimidoacetonitrile group instead of the N-phenyl substitution, stands out as another highly active broad-spectrum agent. It is active against all five bacterial strains, with particularly strong activity against *E. faecalis* and *S. mutans*. The nitrile group is a strong hydrogen bond acceptor, and the entire pyrimidoacetonitrile system may act as a bioisostere for important bacterial enzyme substrates.
- The antioxidant activity, as measured by DPPH radical scavenging, shows a distinct and inverse trend compared to the antibacterial activity, highlighting the divergent structural requirements for these two biological effects. The Pyrimidoacetonitrile moiety is key for potent antioxidant activity: the most potent antioxidant is unequivocally compound 15, which contains the pyrimidoacetonitrile group (44.44% scavenging at 2 mg/mL). This suggests that this specific heterocyclic system is highly effective at donating hydrogen atoms or electrons to stabilize the DPPH radical. Progressive increase in activity with specific core modifications: a clear trend is observed when moving through the synthetic schemes: the initial pyrazolopyridines (1–5) show very low antioxidant activity. The first-generation pyrazolopyridopyrimidines (6–8) show a slight increase. The second-generation pyrazolopyridopyrimidines with various n-substitutions (9–12) show moderate activity. The highest antioxidant activities are found in the final series (13–16), which are structurally distinct from the n-phenyl antibacterial leads. Compounds 13, 14, 15, and 16 are the top antioxidants, with 15 being the most active. The SAR analysis suggests clear structure–activity trends that may guide rational modifications to further enhance potency and selectivity. Future work should focus on in vivo evaluations, pharmacokinetic profiling, and structural optimization to validate clinical relevance and advance these compounds toward potential therapeutic applications.

The molecular docking protocol was rigorously validated through a redocking procedure for each target protein. The native co-crystallized ligand was extracted and re-docked into the defined active site using the same parameters applied to the test compounds. The accuracy was quantified by calculating the Root Mean Square Deviation (RMSD) between the docked and crystallographic poses. As summarized in Table 11, the resulting RMSD values for all targets (0.98–1.58 Å) were significantly below the accepted threshold of 2.0 Å. This confirms the reliability of our docking setup and provides high confidence in the predicted binding poses and interactions for the synthesized compounds. The low RMSD values allow us to state with high confidence that the favorable binding energies, specific hydrogen bonds, and key hydrophobic interactions we report are reliable predictions. This robust validation strongly supports our proposed mechanism of action, wherein the antibacterial activity of lead compounds **7**,** 9**,** 11**,** 12**, and **15** is mediated through the effective inhibition of these essential bacterial targets.

## Experimental section

### Materials and methods

Reagents and chemical used in this study were purchased from commercial sources and used as received. The progress of the chemical reactions was monitored by thin-layer chromatography (TLC). Aluminum sheets and TLC silica gel 60 F 254 (20 × 20 cm) were used for the TLC process. Melting points were determined in open glass capillary tubes on an Electro Thermal Digital melting point apparatus (model: IA9100), and are uncorrected. ^1^H NMR and^13^C NMR spectra were recorded on a Bruker model (300 MHz) Ultra Shield NMR spectrometer in DMSO-d6 using tetramethylsilane (TMS) as an internal standard. Electron impact mass spectra were measured using a DI Analysis Shimadzu QP-2010 plus (70 eV). Elemental analyses were performed using a CHNS-932 (LECO) Vario-Elemental Analyzer. Ortho aminonitrile **3** and compound **16** were synthesized following the previous method^41^. Compounds **4**, **5**, and **14** were prepared following the literature method^47^.

### Synthesis and characterization

#### Prepration of 11-(4-chlorophenyl)-10-methyl-8,11-dihydro-7 H-pyrazolo[4′,3′:5,6]pyrido[3,2-e][1,2,4]triazolo [1,5-c]pyrimidine (**6**)

A mixture of compound **5** (1 mmol) and triethylorthoformate or formic acid (1 mmol) in dry acetic anhydride (5 ml) was heated under reflux for 6 h. The reaction mixture was transferred onto ice (20 gm). The solid formed was collected and recrystallized from Et-OH. Yield: 78%, m.p. 169–171 °C. IR (ν cm^− 1^): 3214, 3120 (2NH); ^1^H NMR δ: 2.03 (s, 3 H, CH_3_), 5.46 (s, 1H, C4-H), 6.26 (s, 1H, NH), 7.31 (d, 2 H, J = 7.6 Hz, Ar-H), 7.46 (d, 2 H, J = 7.6 Hz, Ar-H), 8.31 (s, 1H, Pyrimidine-H), 8.48 (s, 1H, triazole-H), 12.18 (s, 1H, NH); ^13^C NMR (DMSO) δ in ppm: 154.32, 147.70, 142.62, 144.95, 138.00, 134.21, 132.27, 131.72, 131.18, 128.67, 128.51 (2 C), 107.34, 95.26, 36.27, 12.56; MS m/z (%): 338 (21, M + 1). Anal. Calcd for C_16_H_12_ClN_7_: C, 56.90; H, 3.58; Cl, 10.50; N, 29.03. Found: C, 56.93; H, 3.54; Cl, 10.47; N, 29.06.

#### Synthesis of 4-(4-chlorophenyl)-5-imino-3-methyl-N-phenyl-1,4,5,9-tetrahydro-6 H-pyrazolo[4′,3′:5,6]pyrido[2,3-d]pyrimidin-6-amine (**7**)

A mixture of ethyl formimidate **4** (1 mmol) and phenylhydrazine (1.2 mmol) in ethanol (10 mL) was refluxed for 6 h. After cooling, the precipitated solid was filtered and recrystallized from ethanol to yield compound **7**. Yield: 83%, m.p. 157–158 °C. IR (ν cm^− 1^): 3432, 3368, 3210 (4NH). ^1^H NMR (DMSO) δ in ppm: 2.03 (s, 3 H, CH_3_), 4.75 (s, 1H, C4-H), 7.17–7.41 (9 H, Ar-H, 2NH), 8.69 (s, 1H, Pyrimidine-H), 11.40 (s, 1H, NH), 12.11 (s, 1H, NH). MS m/z (%): 404 (11, M + 1). Anal. Calcd for C_21_H_18_ClN_7_: C, 62.45; H, 4.49; Cl, 8.78; N, 24.28. Found: C, 62.48; H, 4.46; Cl, 8.76; N, 24.27.

#### Synthesis of 2,11-Bis(4-chlorophenyl)-10-methyl-8,11-dihydro-7 H-pyrazolo[4′,3′:5,6]pyrido[3,2-e][1,2,4] triazolo[1,5-c]pyrimidine (**8**)

A mixture of compound **5** (1 mmol) and 4-chlorobenzaldehyde (1 mmol) in DMF (5 ml) was heated under reflux for 6 h. The reaction mixture was transferred onto ice (20 gm). The solid formed was collected and recrystallized from EtOH. Yield: 68%, m.p. 149–150 °C. IR (ν cm^− 1^): 3378, 3286 (2NH). ^1^H NMR (DMSO) δ in ppm: 2.15 (s, 3 H, CH_3_), 5.68 (s, 1H, C4-H), 7.33 (d, 2 H, J = 7.6 Hz, Ar-H), 7.35 (d, 2 H, J = 7.6 Hz, Ar-H), 7.36 (d, 2 H, J = 7.6 Hz, Ar-H), 7.56 (d, 2 H, J = 7.6 Hz, Ar-H), 8.40 (s, 1H, Pyrimidine-H), 8.77 (s, 1H, NH), 12.01 (s, 1H, NH). MS m/z (%): 449 (8, M + 1). Anal. Calcd for C_22_H_15_Cl_2_N_7_: C, 58.94; H, 3.37; Cl, 15.81; N, 21.87. Found: C, 58.96; H, 3.35; Cl, 15.80; N, 21.86.

#### Synthetic procedures of 1 H-pyrazolo[4′,3′:5,6]pyrido[2,3-d]pyrimidine derivatives (**9**–**12**)

A mixture of the ethyl formimidate **4** (0.01 mol), and different amines (0.01 mmol) was refluxed in AcOH (10 mL) for 6 h (monitored by TLC). After cooling, the reaction mixture was neutralized using a 10% alcoholic sodium hydroxide solution. The resulting precipitate was filtered, washed thoroughly with water, and recrystallized from acetic acid, yielding the corresponding products (**9**–**12**).

#### N,4-Bis(4-chlorophenyl)-3-methyl-4,9-dihydro-1 H-pyrazolo[4′,3′:5,6]pyrido[2,3-d]pyrimidin-5-amine (**9**)

Yield: 87%, m.p. 213–215 °C. IR (ν cm^− 1^): 3430, 3362, 3196 (3NH). ^1^H NMR (DMSO) δ in ppm: 2.03 (s, 3 H, CH_3_), 5.68 (s, 1H, C4-H), 7.18–7.38 (8 H, Ar-H), 8.36 (s, 1H, Pyrimidine-H), 8.40 (s, 1H, NH), 8.59 (s, 1H, NH), 12.22 (s, 1H, NH); ^13^C NMR δ: 160.16, 155.39, 153.33, 151.41, 145.53, 143.46, 141.36, 135.11, 134.21, 131.72 (2 C), 129.17 (2 C), 128.41 (2 C), 126.93, 118.54, 100.46, 97.19, 35.27, 12.82. MS m/z (%): 423 (8, M + 1). Anal. Calcd for C_21_H_16_Cl_2_N_6_: C, 59.59; H, 3.81; Cl, 16.75; N, 19.85. Found: C, 59.62; H, 3.80; Cl, 16.72; N, 19.86.

#### N-(4-Bromophenyl)-4-(4-chlorophenyl)-3-methyl-4,9-dihydro-1 H-pyrazolo[4′,3′:5,6]pyrido[2,3-d]pyrimidin-5-amine (**10**)

Yield: 84%, m.p. 216–218 °C. IR (ν cm^− 1^): 3426, 3347, 3184 (3NH). ^1^H NMR (DMSO) δ in ppm: 1.91 (s, 3 H, CH_3_), 5.68 (s, 1H, C4-H), 7.33–7.52 (8 H, Ar-H), 8.36 (s, 1H, Pyrimidine-H), 8.40 (s, 1H, NH), 11.88 (s, 1H, NH), 12.22 (s, 1H, NH). MS m/z (%): 467 (16, M + 1). Anal. Calcd for C_21_H_16_BrClN_6_: C, 53.92; H, 3.45; Br, 17.08; Cl, 7.58; N, 17.97. Found: C, 53.95; H, 3.42; Br, 17.07; Cl, 7.56; N, 17.98.

#### 4-(4-Chlorophenyl)-N-(4-methoxyphenyl)-3-methyl-4,9-dihydro-1 H-pyrazolo[4′,3′:5,6]pyrido[2,3-d]pyrimidin-5-amine (**11**)

Yield: 79%, m.p. 223–225 °C. IR (ν cm^− 1^): 3421, 3340, 3182 (3NH). ^1^H NMR (DMSO) δ in ppm: 2.02 (s, 3 H, CH_3_), 3.981(s, 3 H, OCH_3_), 5.58 (s, 1H, C4-H), 7.33–7.34 (2 H, Ar-H), 7.34–7.35 (2 H, Ar-H), 7.35–7.36 (2 H, Ar-H), 7.45–7.47 (2 H, Ar-H), 8.25 (s, 1H, Pyrimidine-H), 8.33 (s, 1H, NH), 11.65 (s, 1H, NH), 12.06 (s, 1H, NH). MS m/z (%): 419 (19, M + 1). Anal. Calcd for C_22_H_19_ClN_6_O: C, 63.08; H, 4.57; Cl, 8.46; N, 20.06; O, 3.82. Found: C, 63.09; H, 4.56; Cl, 8.42; N, 20.08; O, 3.86.

#### 4-((4-(4-Chlorophenyl)-3-methyl-4,9-dihydro-1 H-pyrazolo[4′,3′:5,6]pyrido[2,3-d]pyrimidin-5-yl)amino)-5-methyl-2-phenyl-1,2-dihydro-3 H-pyrazol-3-one (**12**)

Yield: 76%, m.p. 231–232 °C. IR (ν cm^− 1^): 3359, 3268 (4NH), 1643 (C = O). ^1^H NMR (DMSO) δ in ppm: 2.02 (s, 3 H, CH_3_), 2.48 (s, 3 H, CH_3_), 5.46 (s, 1H, C4-H), 7.21–7.52 (8 H, Ar-H), 7.66 (s, 1H, NH), 7.94 (s, 1H, NH), 8.16 (s, 1H, NH), 8.21 (s, 1H, Pyrimidine-H), 12.17 (s, 1H, NH). MS m/z (%): 485 (12, M + 1). Anal. Calcd for C_25_H_21_ClN_8_O: C, 61.92; H, 4.37; Cl, 7.31; N, 23.11; O, 3.30. Found: C, 61.94; H, 4.34; Cl, 7.32; N, 23.10; O, 3.32.

#### Synthesis of 4-(4-chlorophenyl)-3-methyl-1,4,6,9-tetrahydro-5 H-pyrazolo[4′,3′:5,6]pyrido[2,3-d]pyrimidin-5-one (**13**)

A solution of **3** (1mmol) and concentrated HCl (1mL) in glacial acetic acid (5 mL) was refluxed for 12 h. The mixture was poured onto ice and neutralized with NaOH solution. The precipitated formed was filtered and crystallized from ethanol. Yield: 73%, m.p. 263–267 °C. IR (ν cm^− 1^): 3391, 3294, 3190 (3NH), 1620 (C = O).

^1^H NMR (DMSO) δ in ppm: 1.95 (s, 3 H, CH_3_), 4.66 (s, 1H, C4-H), 6.26 (s, 1H, NH), 7.28 (d, 2 H, J = 7.6 Hz, Ar-H), 7.32 (d, 2 H, J = 7.6 Hz, Ar-H), 7.93 (s, 1H, NH), 8.35 (s, 1H, Pyrimidine-H), 10.55 (s, 1H, NH), 10.88 (s, 1H, NH); ^13^C NMR (DMSO) δ in ppm: 159.39, 145.02, 143.02, 140.51, 137.13, 136.14, 134.21, 129.87 (2 C), 128.51 (2 C), 98.27, 92.70, 34.80, 12.16; MS m/z (%): 314 (6, M + 1). Anal. Calcd for C_15_H_12_ClN_5_O: C, 57.42; H, 3.86; Cl, 11.30; N, 22.32; O, 5.10. Found: C, 57.44; H, 3.85; Cl, 11.31; N, 22.30; O, 5.13.

#### Synthesis of 2-(4-(4-chlorophenyl)-3-methyl-5-oxo-4,5,6,9-tetrahydro-1 H-pyrazolo[4′,3′:5,6]pyrido[2,3-d] pyrimidin-7-yl)acetonitrile (**15**)

A mixture of **3** (1 mmol) and ethyl cyanoacetate (1 mmol) in DMF (5mL) was refluxed for 6 h. The mixture was poured onto ice, and precipitate formed was filtered and crystallized from dioxane. Yield: 71%, m.p. 160–162 °C. IR (ν cm^− 1^): 3390, 3291, 3186 (3NH), 1702 (C = O). ^1^H NMR (DMSO) δ in ppm: 1.79 (s, 3 H, CH_3_), 3.86 (s, 2 H, CH_2_), 4.63 (s, 1H, C4-H), 6.26 (s, 1H, NH), 7.18 (d, 2 H, J = 7.6 Hz, Ar-H), 7.36 (d, 2 H, J = 7.6 Hz, Ar-H), 8.15 (s, 1H, Pyrimidine-H), 10.56 (s, 1H, NH), 12.12 (s, 1H, NH). MS m/z (%): 353 (11, M + 1). Anal. Calcd for C_17_H_13_ClN_6_O: C, 57.88; H, 3.71; Cl, 10.05; N, 23.82; O, 4.54. Found: C, 57.89; H, 3.69; Cl, 10.06; N, 23.84; O, 4.56.

### Evaluation of antioxidant activity of compounds

#### DPPH radical-scavenging assay

The free radical-scavenging activity of the synthesized compounds was evaluated using the 2,2-diphenyl-1-picrylhydrazyl (DPPH) assay, following the method of (Mansoor et al. 2022)^49^ 494,949 with slight modifications. A freshly prepared 0.3 mm DPPH solution in methanol was used for the assay. An aliquot of the test compound solution (1.0 mL) was mixed with an equal volume of the DPPH solution (1.0 mL), vortexes briefly, and incubated in the dark at 30 °C for 30 min. Absorbance at 517 nm was measured using a UV–Vis spectrophotometer. The radical-scavenging activity was expressed as the percentage inhibition of DPPH, calculated according to the equation:

$${\text{Scavenging activity }}\left( \% \right) = \left[ {\left( {\frac{{{\mathrm{Control}}\;{\mathrm{absorbance}} - {\mathrm{sample}}\;{\mathrm{absorbance}}}}{{{\mathrm{absorbance}}\;{\mathrm{of}}\;{\mathrm{control}}}}} \right) \times 100\% } \right]$$

#### Assessment of a compound’s antibacterial activity

All compound were screened against pathogenic bacterial strains (Escherichia coli, Staphylococcus aureus, S. mutans, Enterococcus faecalis and Pseudomonas aeruginosa) using the agar well diffusion method by (Magaldi et al., 2004)^50^. All strains were obtained from the Microbial Genetics Lab., National Research Centre, Egypt. Nutrient broth was used to sub-culture the investigated microbes, and then incubated for 24 h. at 37 °C and 120 rpm. Each strain was swabbed with sterile cotton swabs on Mueller-Hinton agar. Also, 100 µl of compounds were inoculated to their wells. After 24 h at 37 °C, the zone of inhibition was evaluated using a zone scale.

### Molecular socking

Table 11 provided all of the protein receptors. This was followed by a pretreatment step utilizing PyMOL software to remove existing ligands, ions, and water molecules from the target protein structures. The compound’s structure was drawn using BIOVIA draw. Then, open Babel^51^was utilized to convert each compound into the mol2 format. Subsequently, autodock tools were used to convert the molecules into the pdbqt format. Prior to docking, Ligand-centered maps were generated using autodock Vina^52^. Discovery Studio program was employed to analyze the 2-D interactions between the target and the ligands. The physicochemical parameters and ADMET of compounds were calculated using the BIOVIA Discovery Studio software^53^ The molecular docking protocol was validated through a redocking procedure for each target protein. The native co-crystallized ligand was extracted and re-docked into the defined active site using the same parameters applied to the test compounds. The accuracy was quantified by calculating the Root Mean Square Deviation (RMSD) between the docked and crystallographic poses.

**Table 11:** PDB IDs, active site coordinates native co-crystallized ligands, reference ligands, and molecular Docking targets of bacterial strains.

Organism	Protein targets	PDB ID	Resolution (Å)	Active site coordinates:			Native ligands	RMSD values	Common residues
				X	Y	Z			
*E. faecalis*	KPC-2 carbapenemase	2OV5	1.85	40.3 (60 Å)	1.52 (60 Å)	8.17 (60 Å)	BCN	1.25 Å	Glu276 Thr216 Thr235
*E.coli*	DNA Gyrase	7P2M	1.16	4.36 (60 Å)	33.47 (60 Å)	− 14.16 (60 Å)	N1N	1.58 Å	Asp73 Gly77 Thr165
*S. aureus*	Dihydropteroate synthase	1AD4	2.40	33.4 (60 Å)	5.95 (60 Å)	37.9 (60 Å)	HH2	0.98 Å	Arg52 His55 Val49
*P. aeruginosa*	LasR an activator of exotoxin A expression	2UV0	1.80	24.37 (60 Å)	13.79 (60 Å)	81.52 (60 Å)	OHN	1.42 Å	Thr75 Glu48 Ser129

## Conclusion

This study focuses on synthesizing fused pyrazolopyridine analogues to serve as antibacterial and antioxidant agents. The targeted compounds underwent synthesis and characterization through elemental and spectrometric analyses. Following this, The antioxidant and antibacterial properties of the recently synthesized compounds were assessed, investigating their interactions with crucial proteins using molecular docking. Notably, compounds **7**, **9**, **11**, **12**, and **15** showed strong antibacterial properties against a range of bacterial species, such as S. aureus, E. coli, E. fecalis, P. aeruginosa, and S. mutans. Additionally, using DPPH and ABTS radical scavenging techniques, their antioxidant qualities were evaluated. Compound 15 demonstrated outstanding DPPH radical scavenging capabilities. Favorable binding energies were found by molecular docking screens, indicating the compounds’ potential to efficiently control antioxidant enzymes. Moreover, in-silico ADMET and drug-likeness screenings indicated that compounds **7**, **9**, **11**, and **15** adhere to the Lipinski rules, highlighting their promise as potential orally bioavailable drug candidates with favorable properties. The findings indicate that these substances may be further refined and improved as antioxidants and antimicrobials. Highlighting these next steps emphasizes the potential of this work to contribute to the discovery of new therapeutic agents addressing urgent medical needs, particularly in the fight against antimicrobial resistance.

## Supplementary Information

Below is the link to the electronic supplementary material.

Supplementary Material 1

## Data Availability

All data generated or analyzed are included in this published article and its supplementary information file.
