# Supplementary material for: Synthesis, molecular docking, ADMET studies and biological evaluation of fused pyrazolopyridopyrimidine derivatives as antioxidant and antimicrobial agents
Source: Sci Rep. 2025 Dec 11;15:43654. doi: 10.1038/s41598-025-30217-9 (PMC12701012; doi:10.1038/s41598-025-30217-9)

**Synthesis and Biological Assessment of Fused pyrazolopyridopyrimidine analogues: Antioxidant Activity, Antimicrobial, Molecular Docking, and ADMET studies**

Experimental Section

**General**

All chemicals were purchased from Sigma-Aldrich and were used as obtained without further purification. The progress of the chemical reactions was monitored by thin-layer chromatography (TLC). TLC was performed on aluminum sheets, TLC silica gel 60 F 254 (20 ×20 cm). Melting points were determined in open glass capillary tubes on an Electro Thermal Digital melting point apparatus (model: IA9100), and are uncorrected. ^1^H NMR and ^13^C NMR spectra were recorded on a Bruker model (300 MHz) Ultra Shield NMR spectrometer in DMSO-d6 using tetramethylsilane (TMS) as an internal standard. Electron impact mass spectra were measured using a DI Analysis Shimadzu QP-2010 plus (70 eV). Elemental analyses were performed using a CHNS-932 (LECO) Vario-Elemental Analyzer. Ortho aminonitrile **3** and compound **16** were synthesized following the previous method. Compounds **4**, **5**, and **14** were prepared following the literature method.

**Synthesis of 11-(4-chlorophenyl)-10-methyl-8,11-dihydro-7H-pyrazolo[4',3':5,6]pyrido[3,2-e][1,2,4]triazolo[1,5-c]pyrimidine** (**6).**

A mixture of compound **5** (1 mmol) and triethylorthoformate or formic acid (1 mmol) in dry acetic anhydride (5 ml) was heated under reflux for 6 hrs. The reaction mixture was transferred onto ice (20 gm). The solid formed was collected and recrystallized from EtOH. Yield: 78%, m.p. 169–171°C. **IR**: 3214, 3120 (2NH); **^1^H NMR** δ: 2.03 (s, 3H, CH_3_), 5.46 (s, 1H, C4-H), 6.26 (s, 1H, NH), 7.31 (d, 2H, *J* =7.6Hz, Ar-H), 7.46 (d, 2H, *J* =7.6Hz, Ar-H), 8.31 (s, 1H, Pyrimidine-H), 8.48 (s, 1H, triazole-H), 12.18 (s, 1H, NH); **^13^C NMR**: δ 154.32, 147.70, 142.62, 144.95, 138.00, 134.21, 132.27, 131.72, 131.18, 128.67, 128.51 (2C), 107.34, 95.26, 36.27, 12.56; MS m/z (%): 338 (21, M+1). Anal. Calcd for C_16_H_12_ClN_7_: C, 56.90; H, 3.58; Cl, 10.50; N, 29.03. Found: C, 56.93; H, 3.54; Cl, 10.47; N, 29.06.

**Synthesis of 4-(4-chlorophenyl)-5-imino-3-methyl-N-phenyl-1,4,5,9-tetrahydro-6H-pyrazolo[4',3':5,6]pyrido[2,3-d]pyrimidin-6-amine (7).**

A mixture of ethyl formimidate **4** (1 mmol) and phenylhydrazine (1.2 mmol) in ethanol (10 mL) was refluxed for 6 hours. After cooling, the precipitated solid was filtered and recrystallized from ethanol to yield compound **7**. Yield: 83%, m.p. 157–158°C. **IR**: IR: 3432, 3368, 3210 (4NH). **^1^H NMR** δ: 2.03 (s, 3H, CH_3_), 4.75 (s, 1H, C4-H), 7.17–7.41 (9H, Ar-H, 2NH), 8.69 (s, 1H, Pyrimidine-H), 11.40 (s, 1H, NH), 12.11 (s, 1H, NH). MS m/z (%): 404 (11, M+1). Anal. Calcd for C_21_H_18_ClN_7_: C, 62.45; H, 4.49; Cl, 8.78; N, 24.28. Found: C, 62.48; H, 4.46; Cl, 8.76; N, 24.27.

**Synthesis of 2,11-Bis(4-chlorophenyl)-10-methyl-8,11-dihydro-7H-pyrazolo[4',3':5,6]pyrido[3,2-e][1,2,4]triazolo[1,5-c]pyrimidine** (**8).**

A mixture of compound **5** (1 mmol) and 4-chlorobenzaldehyde (1 mmol) in DMF (5 ml) was heated under reflux for 6 hrs. The reaction mixture was transferred onto ice (20 gm). The solid formed was collected and recrystallized from EtOH. Yield: 68%, m.p. 149–150°C. **IR**: 3378, 3286 (2NH). **^1^H NMR** δ: 2.15 (s, 3H, CH_3_), 5.68 (s, 1H, C4-H), 7.33 (d, 2H, *J* =7.6Hz, Ar-H), 7.35 (d, 2H, *J*=7.6Hz, Ar-H), 7.36 (d, 2H, *J* =7.6Hz, Ar-H), 7.56 (d, 2H, *J*=7.6Hz, Ar-H), 8.40 (s, 1H, Pyrimidine-H), 8.77 (s, 1H, NH), 12.01 (s, 1H, NH). MS m/z (%): 449 (8, M+1). Anal. Calcd for C_22_H_15_Cl_2_N_7_: C, 58.94; H, 3.37; Cl, 15.81; N, 21.87. Found: C, 58.96; H, 3.35; Cl, 15.80; N, 21.86.

**Synthetic procedures of 1H-pyrazolo[4',3':5,6]pyrido[2,3-d]pyrimidine derivatives (9-12).**

A mixture of the ethyl formimidate **4** (0.01 mol), and different amines (0.01 mmol) was refluxed in AcOH (10 mL) for 6 hours (monitored by TLC). After cooling, the reaction mixture was neutralized using a 10% alcoholic sodium hydroxide solution. The resulting precipitate was filtered, washed thoroughly with water, and recrystallized from acetic acid, yielding the corresponding products (**9**-**12**).

**N,4-Bis(4-chlorophenyl)-3-methyl-4,9-dihydro-1H-pyrazolo[4',3':5,6]pyrido[2,3-d]pyrimidin-5-amine (9).**

Yield: 87%, m.p. 213–215°C. **IR**: IR: 3430, 3362, 3196 (3NH). **^1^H NMR** δ: 2.03 (s, 3H, CH_3_), 5.68 (s, 1H, C4-H), 7.18–7.38 (8H, Ar-H), 8.36 (s, 1H, Pyrimidine-H), 8.40 (s, 1H, NH), 8.59 (s, 1H, NH), 12.22 (s, 1H, NH); **^13^C NMR** δ: 160.16, 155.39, 153.33, 151.41, 145.53, 143.46, 141.36, 135.11, 134.21, 131.72 (2C), 129.17 (2C), 128.41 (2C), 126.93, 118.54, 100.46, 97.19, 35.27, 12.82. MS m/z (%): 423 (8, M+1). Anal. Calcd for C_21_H_16_Cl_2_N_6_: C, 59.59; H, 3.81; Cl, 16.75; N, 19.85. Found: C, 59.62; H, 3.80; Cl, 16.72; N, 19.86.

**N-(4-Bromophenyl)-4-(4-chlorophenyl)-3-methyl-4,9-dihydro-1H-pyrazolo[4',3':5,6]pyrido[2,3-d]pyrimidin-5-amine (10).**

Yield: 84%, m.p. 216–218°C. **IR**: IR: 3426, 3347, 3184 (3NH). **^1^H NMR** δ: 1.91 (s, 3H, CH_3_), 5.68 (s, 1H, C4-H), 7.33–7.52 (8H, Ar-H), 8.36 (s, 1H, Pyrimidine-H), 8.40 (s, 1H, NH), 11.88 (s, 1H, NH), 12.22 (s, 1H, NH). MS m/z (%): 467 (16, M+1). Anal. Calcd for C_21_H_16_BrClN_6_: C, 53.92; H, 3.45; Br, 17.08; Cl, 7.58; N, 17.97. Found: C, 53.95; H, 3.42; Br, 17.07; Cl, 7.56; N, 17.98.

**4-(4-Chlorophenyl)-N-(4-methoxyphenyl)-3-methyl-4,9-dihydro-1H-pyrazolo[4',3':5,6]pyrido[2,3-d]pyrimidin-5-amine (11).**

Yield: 79%, m.p. 223–225°C. IR: **IR**: 3421, 3340, 3182 (3NH). **^1^H NMR** δ: 2.02 (s, 3H, CH_3_), 3.981(s, 3H, OCH_3_), 5.58 (s, 1H, C4-H), 7.33–7.34 (2H, Ar-H), 7.34–7.35 (2H, Ar-H), 7.35–7.36 (2H, Ar-H), 7.45–7.47 (2H, Ar-H), 8.25 (s, 1H, Pyrimidine-H), 8.33 (s, 1H, NH), 11.65 (s, 1H, NH), 12.06 (s, 1H, NH). MS m/z (%): 419 (19, M+1). Anal. Calcd for C_22_H_19_ClN_6_O: C, 63.08; H, 4.57; Cl, 8.46; N, 20.06; O, 3.82. Found: C, 63.09; H, 4.56; Cl, 8.42; N, 20.08; O, 3.86.

**4-((4-(4-Chlorophenyl)-3-methyl-4,9-dihydro-1H-pyrazolo[4',3':5,6]pyrido[2,3-d]pyrimidin-5-yl)amino)-5-methyl-2-phenyl-1,2-dihydro-3H-pyrazol-3-one (12).**

Yield: 76%, m.p. 231–232°C. **IR**: IR: 3359, 3268 (4NH), 1643 (C=O). **^1^H NMR** δ: 2.02 (s, 3H, CH_3_), 2.48 (s, 3H, CH_3_), 5.46 (s, 1H, C4-H), 7.21–7.52 (8H, Ar-H), 7.66 (s, 1H, NH), 7.94 (s, 1H, NH), 8.16 (s, 1H, NH), 8.21 (s, 1H, Pyrimidine-H), 12.17 (s, 1H, NH). MS m/z (%): 485 (12, M+1). Anal. Calcd for C_25_H_21_ClN_8_O: C, 61.92; H, 4.37; Cl, 7.31; N, 23.11; O, 3.30. Found: C, 61.94; H, 4.34; Cl, 7.32; N, 23.10; O, 3.32.

**Synthesis of** **4-(4-chlorophenyl)-3-methyl-1,4,6,9-tetrahydro-5H-pyrazolo[4',3':5,6]pyrido[2,3-d]pyrimidin-5-one (13).**

A solution of **3** (1mmol) and concentrated HCl (1mL) in glacial acetic acid (5 mL) was refluxed for 12h. The mixture was poured onto ice and neutralized with NaOH solution. The precipitated formed was filtered and crystallized from ethanol. Yield: 73%, m.p. 263–267°C. **IR**: 3391, 3294, 3190 (3NH), 1620 (C=O). **^1^H NMR** δ: 1.95 (s, 3H, CH_3_), 4.66 (s, 1H, C4-H), 6.26 (s, 1H, NH), 7.28 (d, 2H, J =7.6Hz, Ar-H), 7.32 (d, 2H, J=7.6Hz, Ar-H), 7.93 (s, 1H, NH), 8.35 (s, 1H, Pyrimidine-H), 10.55 (s, 1H, NH), 10.88 (s, 1H, NH); **^13^C NMR** δ: 159.39, 145.02, 143.02, 140.51, 137.13, 136.14, 134.21, 129.87 (2C), 128.51 (2C), 98.27, 92.70, 34.80, 12.16; MS m/z (%): 314 (6, M+1). Anal. Calcd for C_15_H_12_ClN_5_O: C, 57.42; H, 3.86; Cl, 11.30; N, 22.32; O, 5.10. Found: C, 57.44; H, 3.85; Cl, 11.31; N, 22.30; O, 5.13.

**Synthesis of 2-(4-(4-chlorophenyl)-3-methyl-5-oxo-4,5,6,9-tetrahydro-1H-pyrazolo[4',3':5,6]pyrido[2,3-d]pyrimidin-7-yl)acetonitrile (15).**

A mixture of **3** (1 mmol) and ethyl cyanoacetate (1 mmol) in DMF (5mL) was refluxed for 6h. The mixture was poured onto ice, and precipitate formed was filtered and crystallized from dioxane. Yield: 71%, m.p. 160–162°C. **IR**: 3390, 3291, 3186 (3NH), 1702 (C=O). **^1^H NMR** δ: 1.79 (s, 3H, CH_3_), 3.86 (s, 2H, CH_2_), 4.63 (s, 1H, C4-H), 6.26 (s, 1H, NH), 7.18 (d, 2H, J =7.6Hz, Ar-H), 7.36 (d, 2H, J=7.6Hz, Ar-H), 8.15 (s, 1H, Pyrimidine-H), 10.56 (s, 1H, NH), 12.12 (s, 1H, NH). MS m/z (%): 353 (11, M+1). Anal. Calcd for C_17_H_13_ClN_6_O: C, 57.88; H, 3.71; Cl, 10.05; N, 23.82; O, 4.54. Found: C, 57.89; H, 3.69; Cl, 10.06; N, 23.84; O, 4.56.

## Evaluation of antioxidant activity of compounds:

### **DPPH free radical scavenging activity.**

According to (Mansoor et al., 2022) DPPH was used to study the free radical-scavenging activities of compounds. one ml of the compounds was added to 1.0 ml methanolic solution of 0.3 mM DPPH. The mixture was shaken and left in a dark box for 30 minutes at room temperature (30°C). The absorbance of the resulting solution was measured at 517 nm. The inhibitory percentage of DPPH was calculated according to the following equation:

Scavenging activity (%) = [ ($\frac{Control absorbance - sample absorbance)}{absorbance of control}$) X 100%]

## Evaluation of antibacterial activity of compounds

### All compound were screened against pathogenic bacterial strains (*Escherichia coli*, *Staphylococcus aureus*, *S. mutans*, *Enterococcus faecalis* and *Pseudomonas aeruginosa*) using the agar well diffusion method by (Magaldi et al., 2004). All strains were obtained from the Microbial Genetics Lab., National Research Centre, Egypt. Nutrient broth was used to sub-culture the investigated microbes, then incubated for 24 hrs at 37°C and 120 rpm. Each strain was swabbed with sterile cotton swabs on Mueller-Hinton agar. Also, 100µl of compounds were inoculated to their wells. After 24 hours at 37 °C, the zone of inhibition was evaluated using a zone scale.

## Computational methods

**Molecular Docking**

All protein receptors were acquired from the RCSB **Table 10**. Subsequently, the structures of the target proteins underwent a preprocessing step using PyMOL software, which involved the removal of water molecules, ions, and existing ligands. The compound’s structure was drawn using BIOVIA draw. Then, open Babel **(O'Boyle et al., 2011)** was utilized to convert each compound into the mol2 format. Subsequently, autodock tools were used to convert the molecules into the pdbqt format. Prior to docking, Ligand-centered maps were generated using autodock Vina **(Eberhardt et al., 2021).** Discovery Studio program was employed to analyze the 2-D interactions between the target and the ligands. The physicochemical parameters and ADMET of compounds were calculated using the BIOVIA Discovery Studio software (**Daina, A. & Zoete, V).**


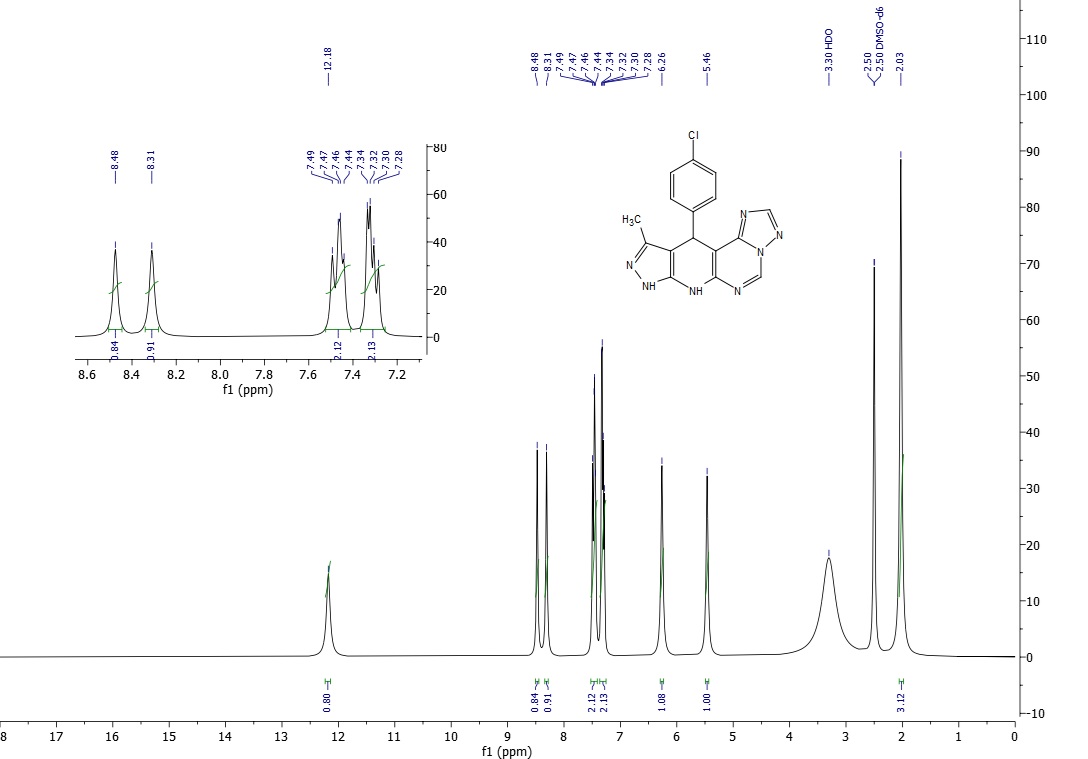


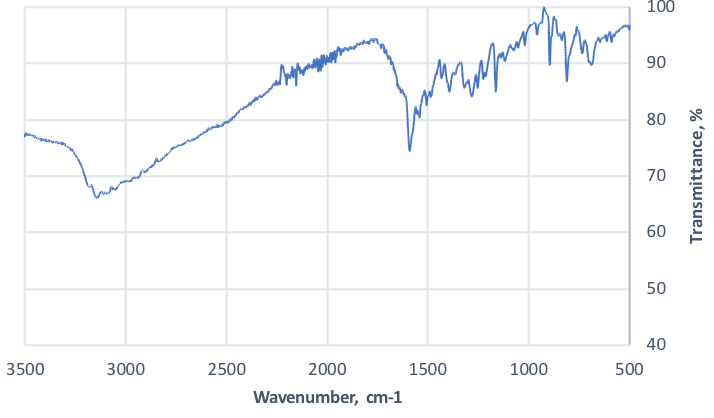


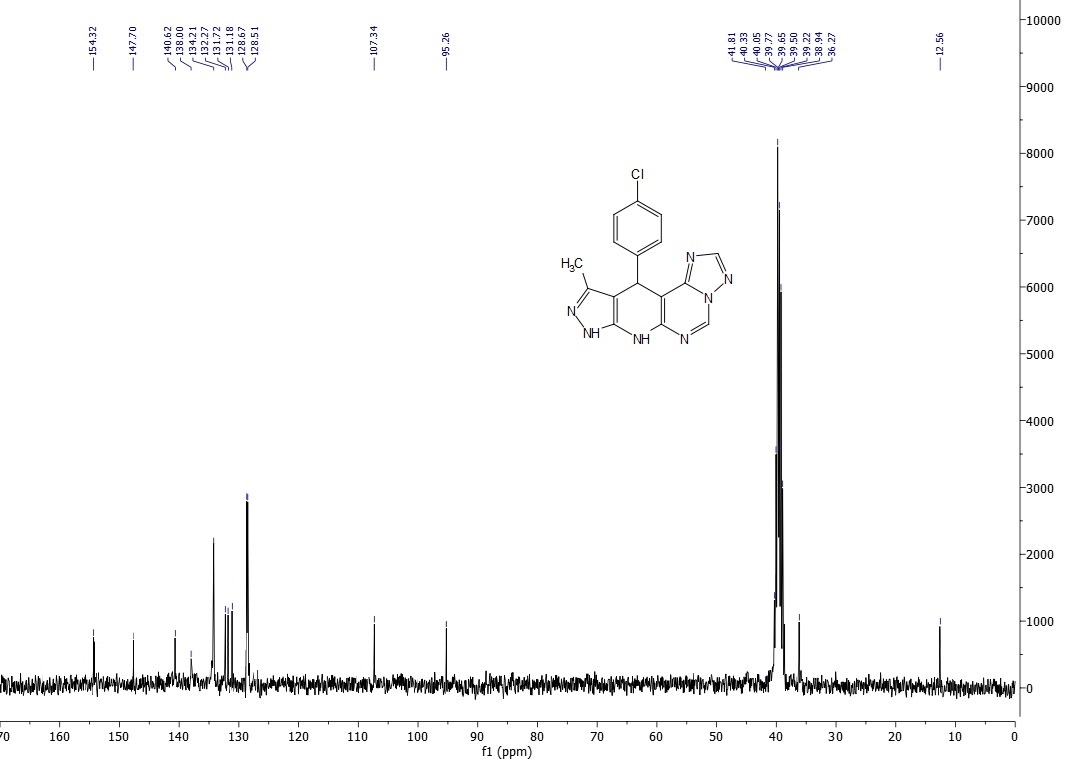


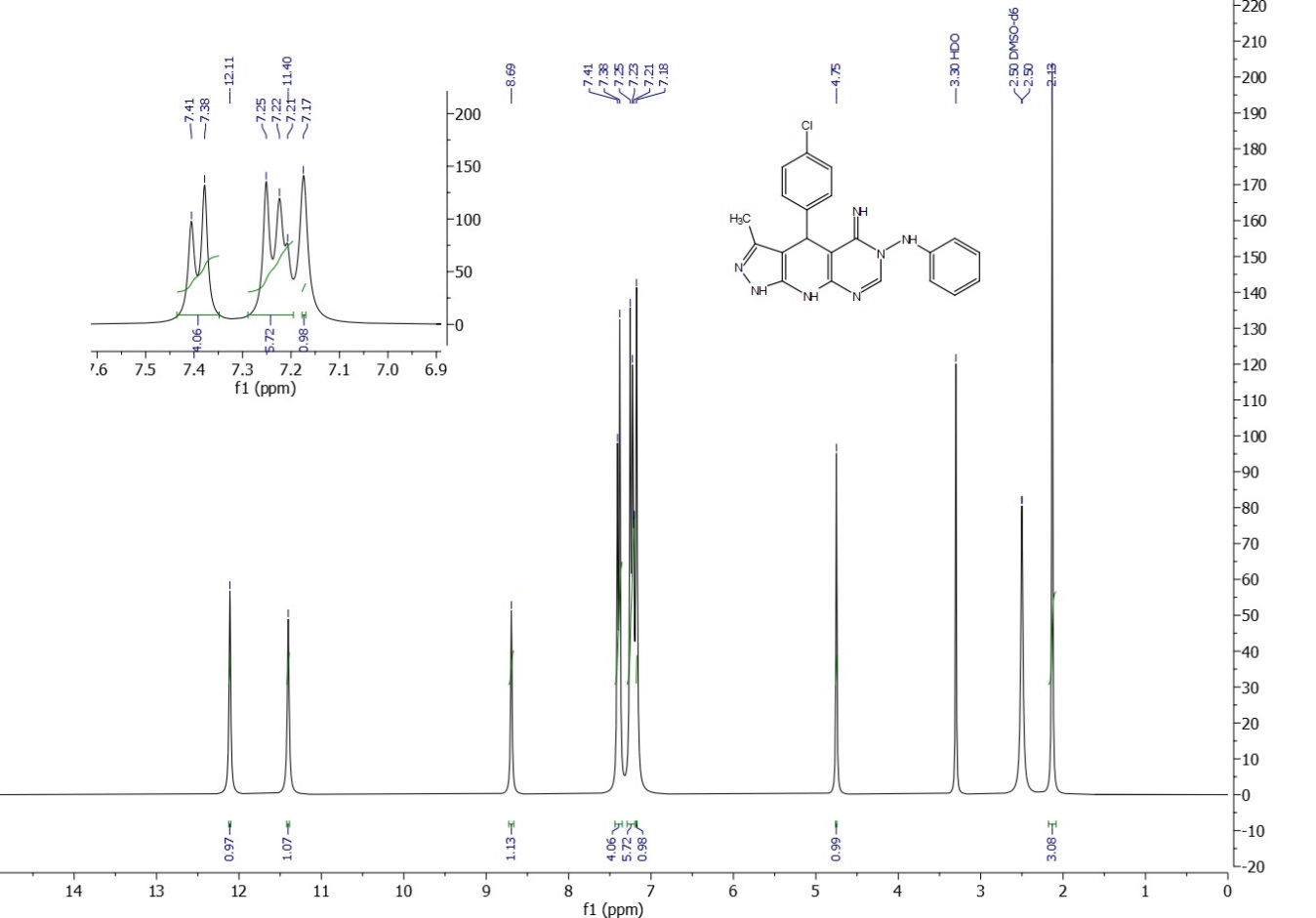


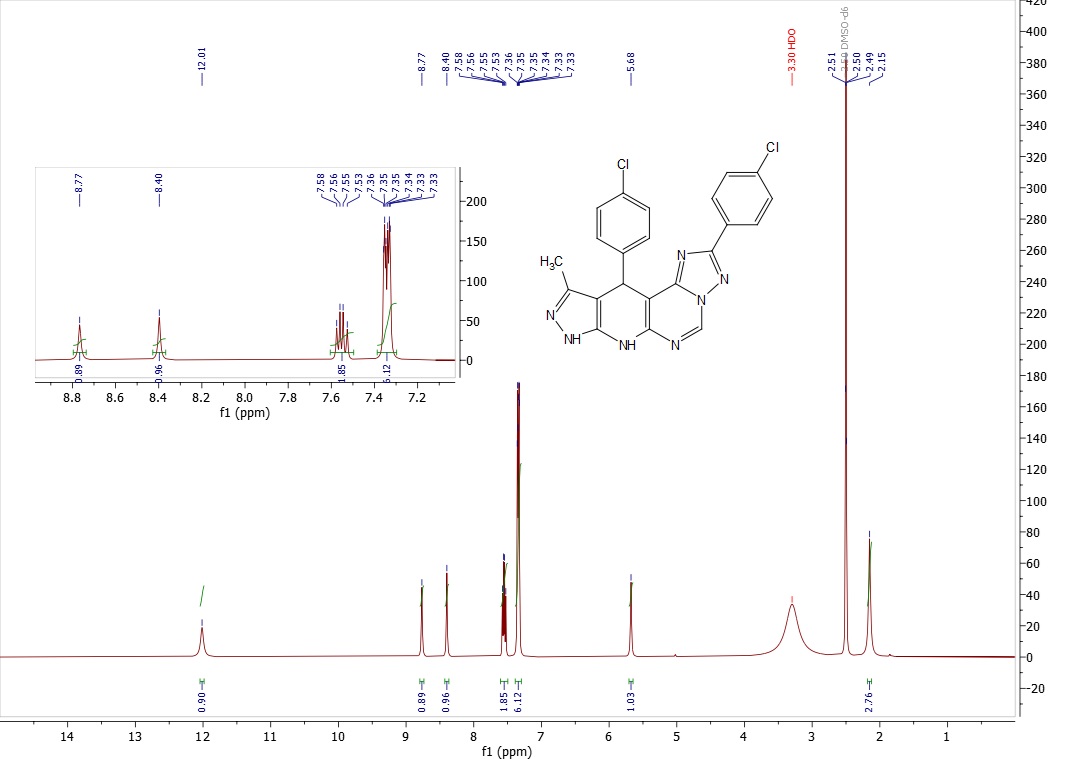


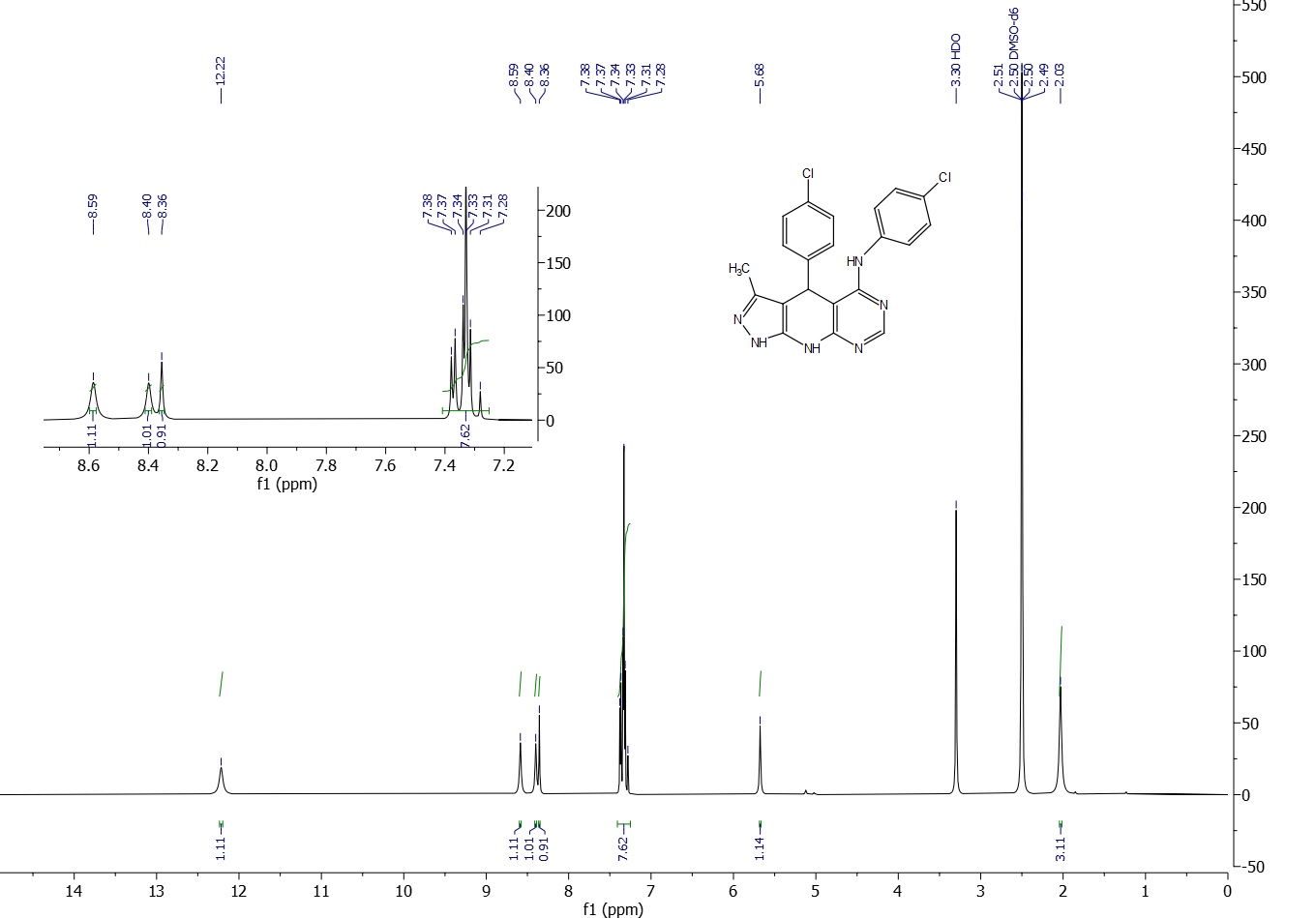


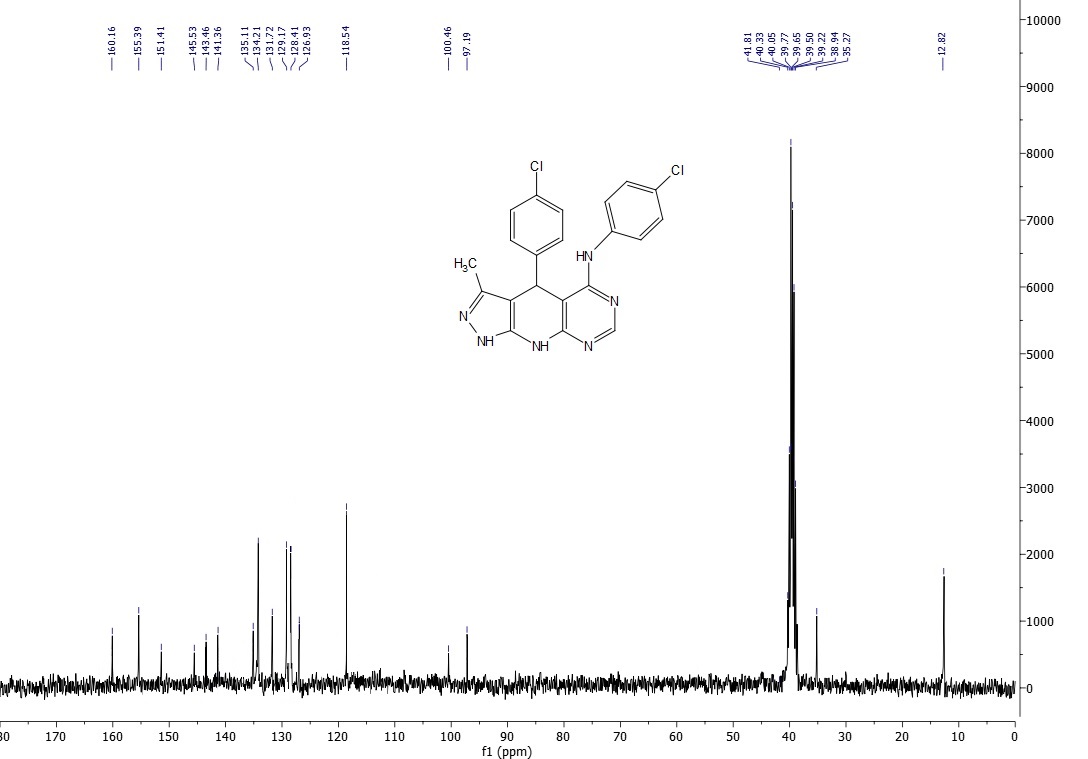


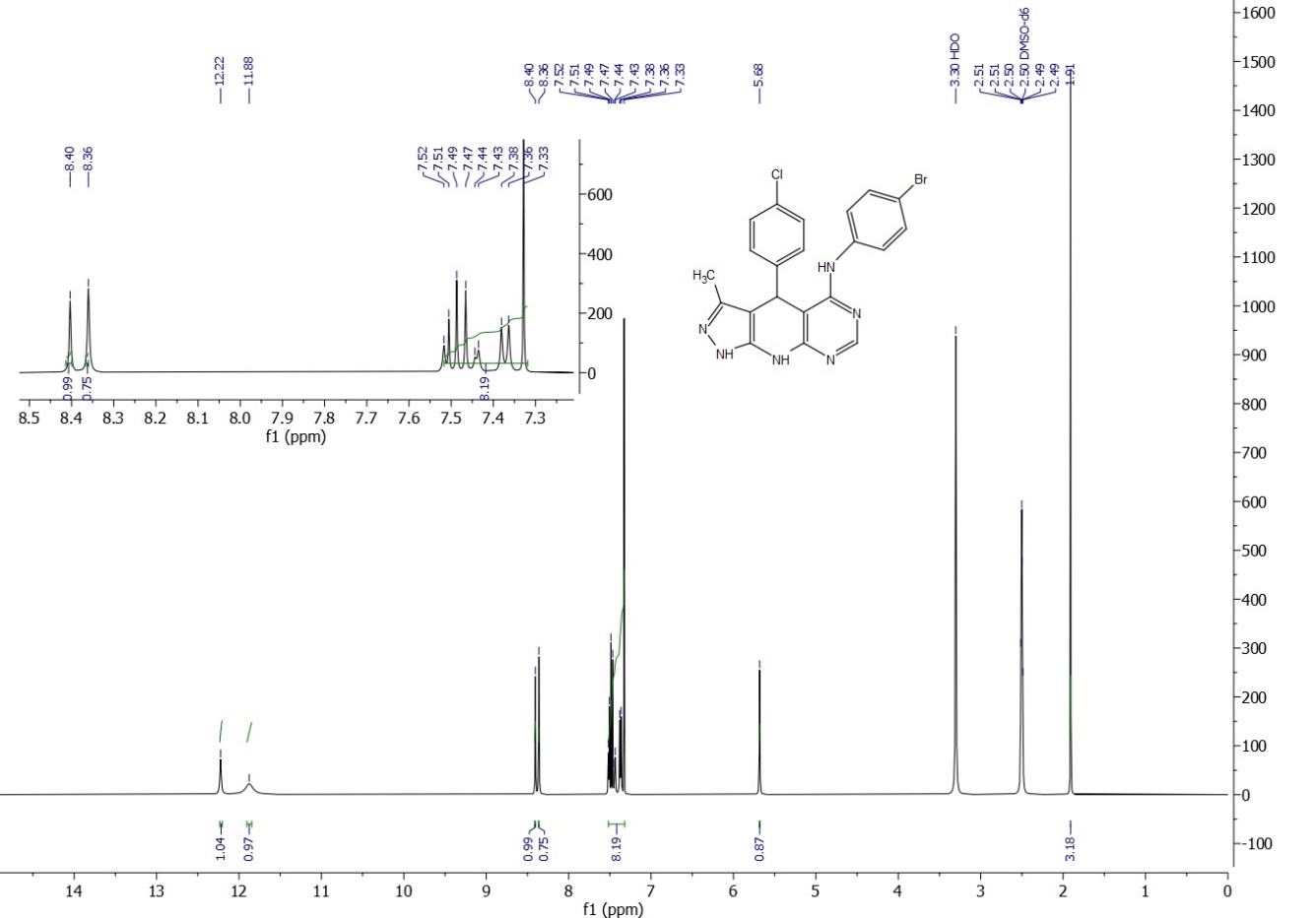


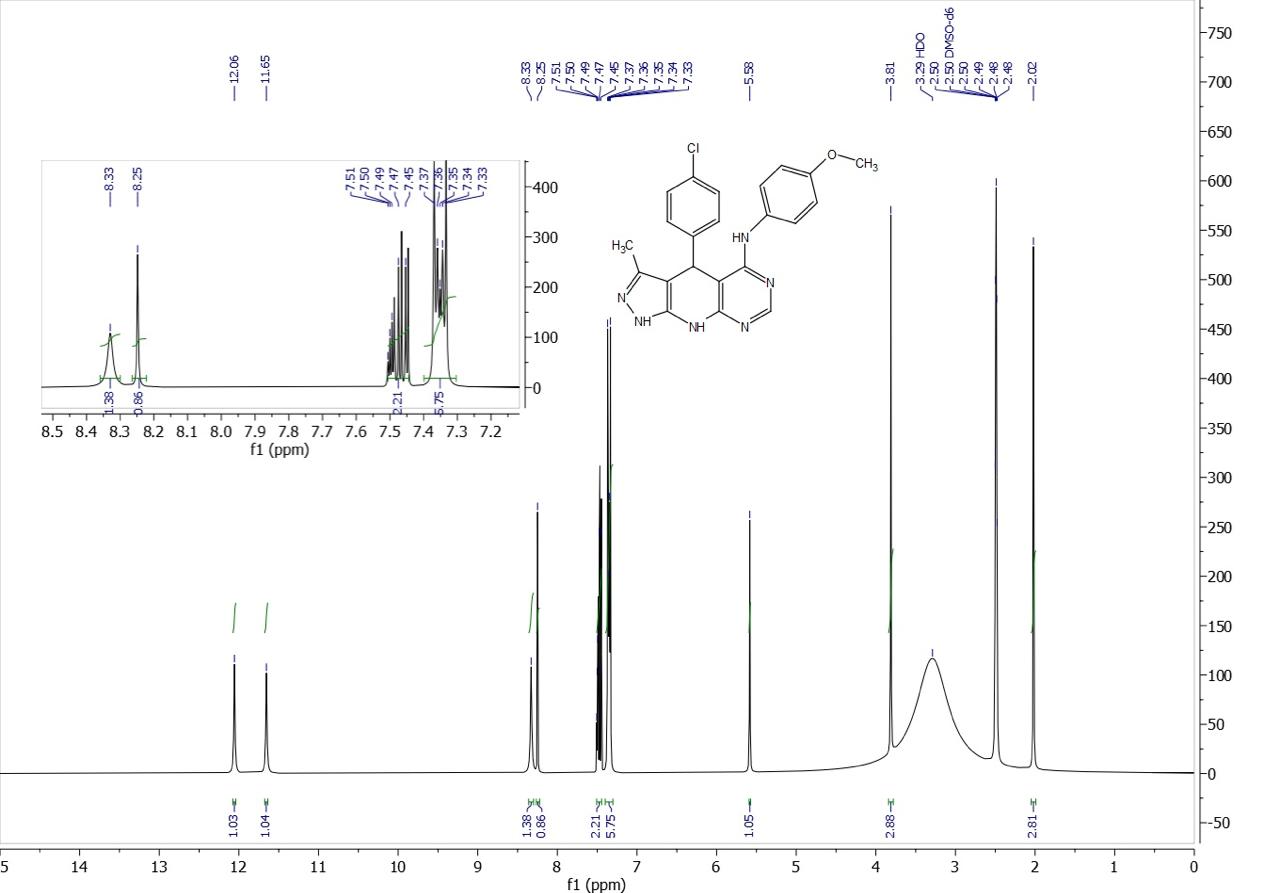


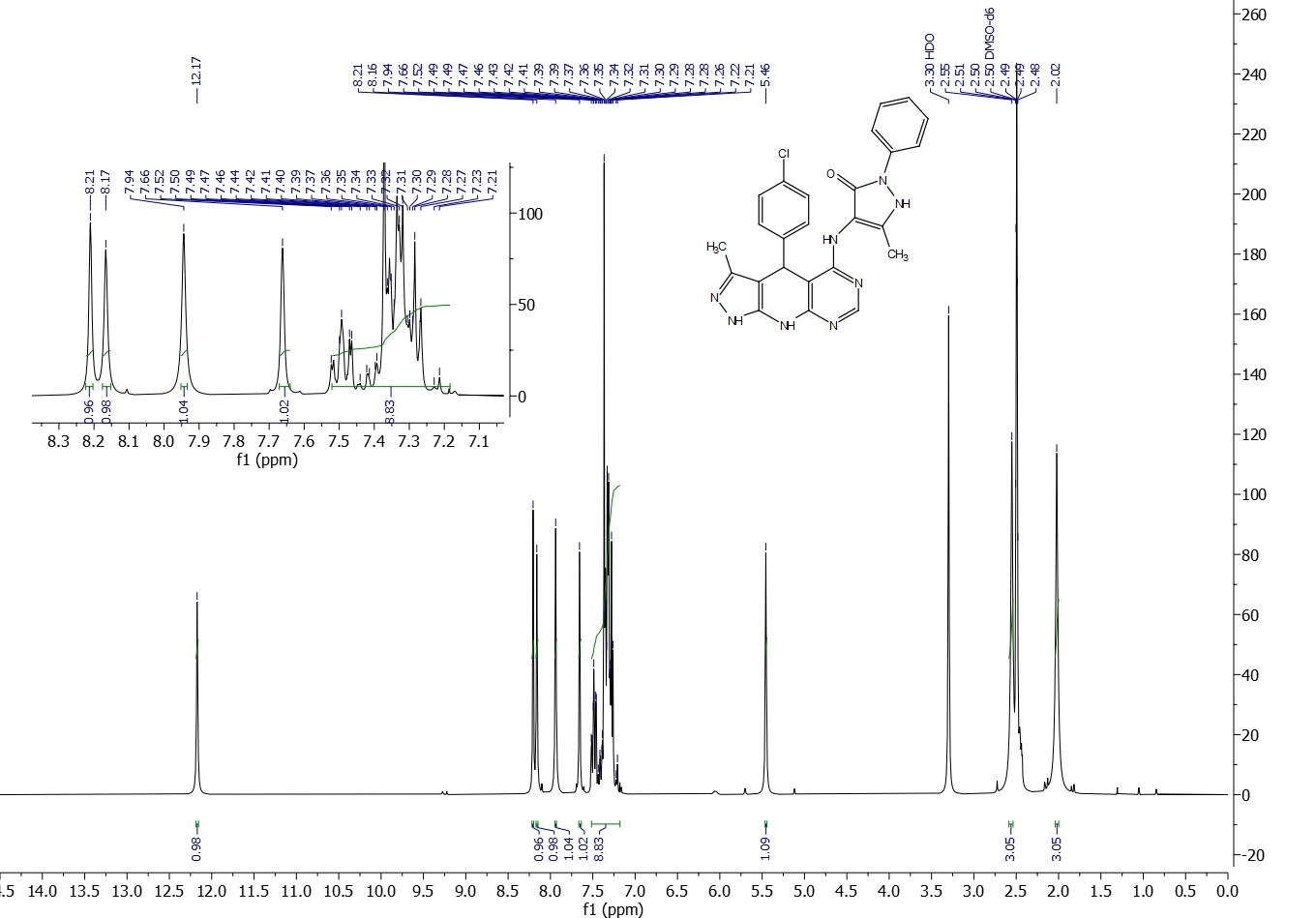


**
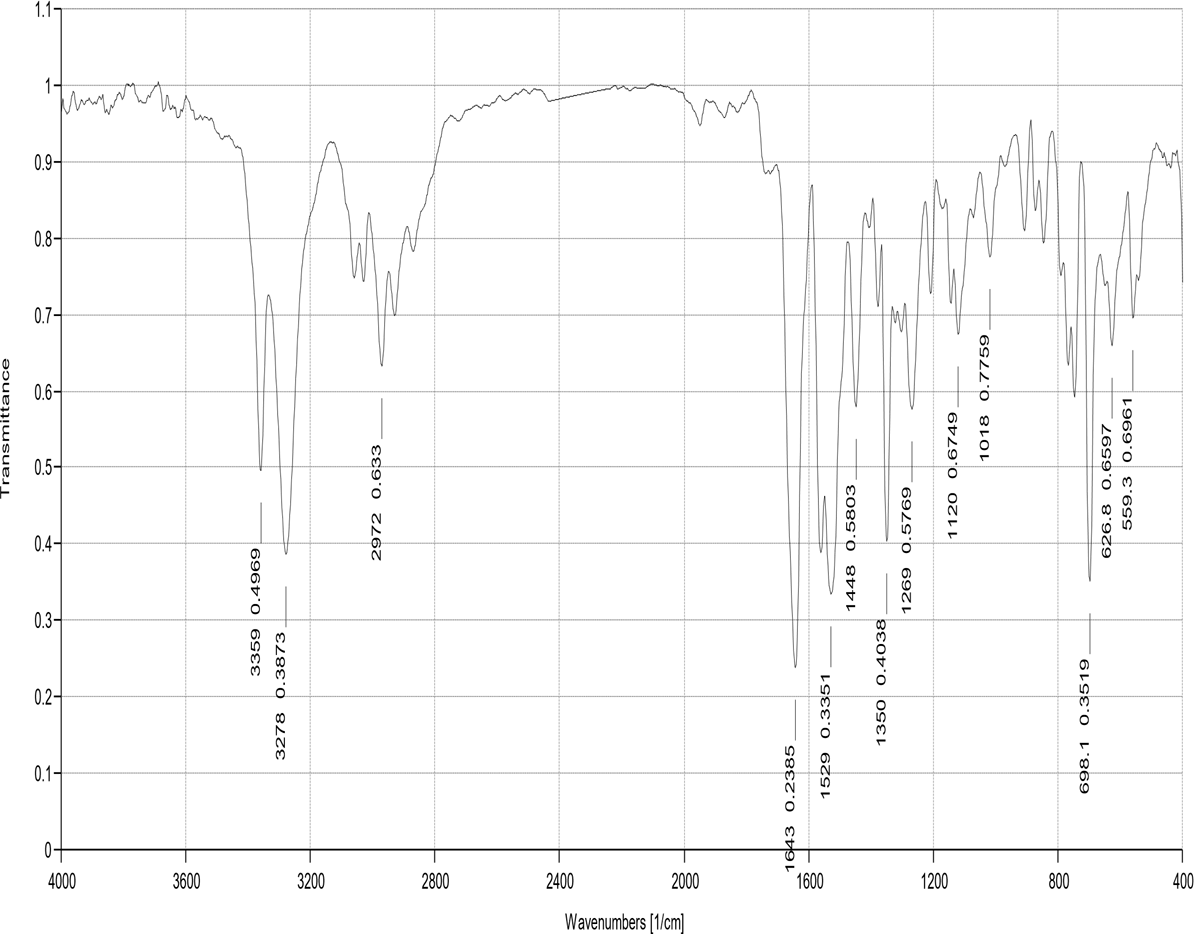
**


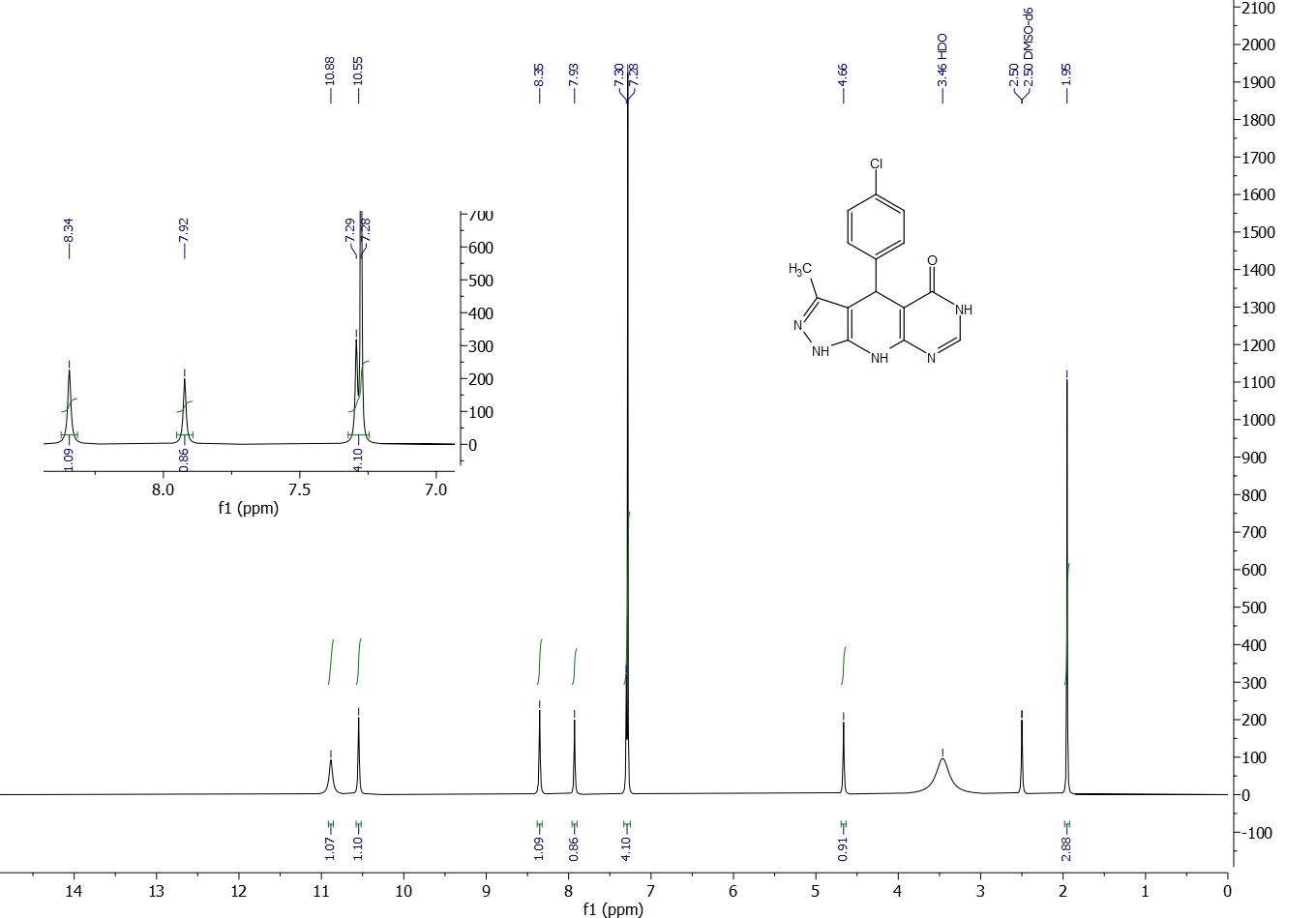


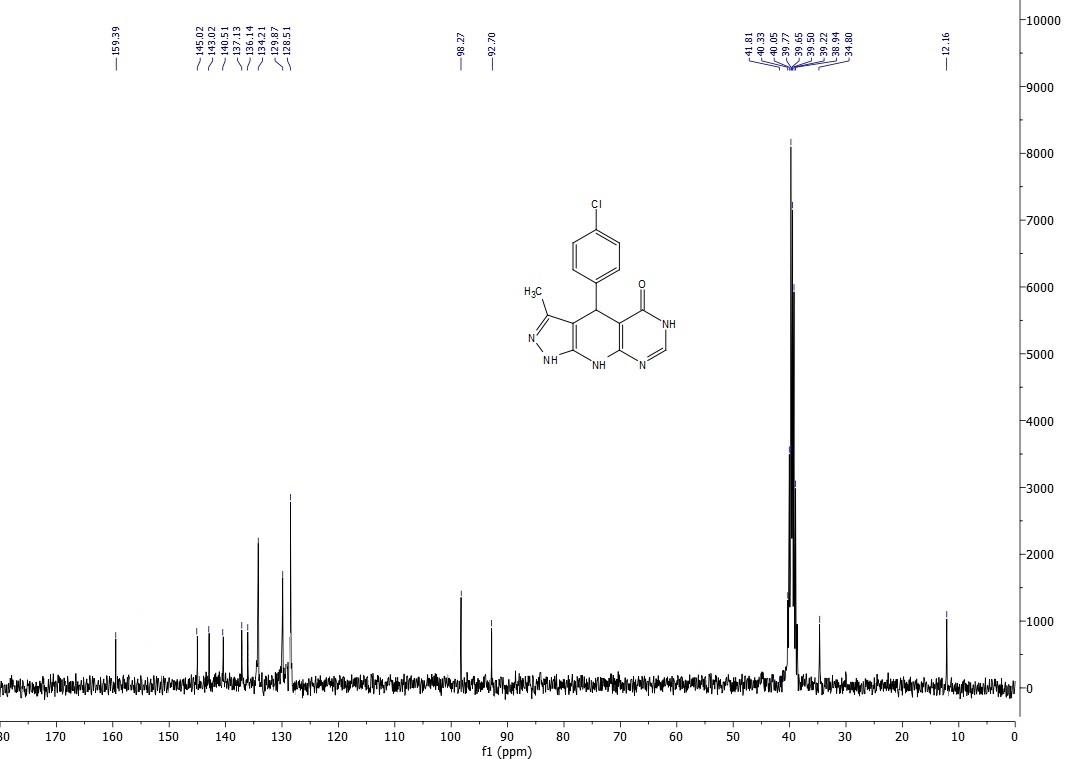


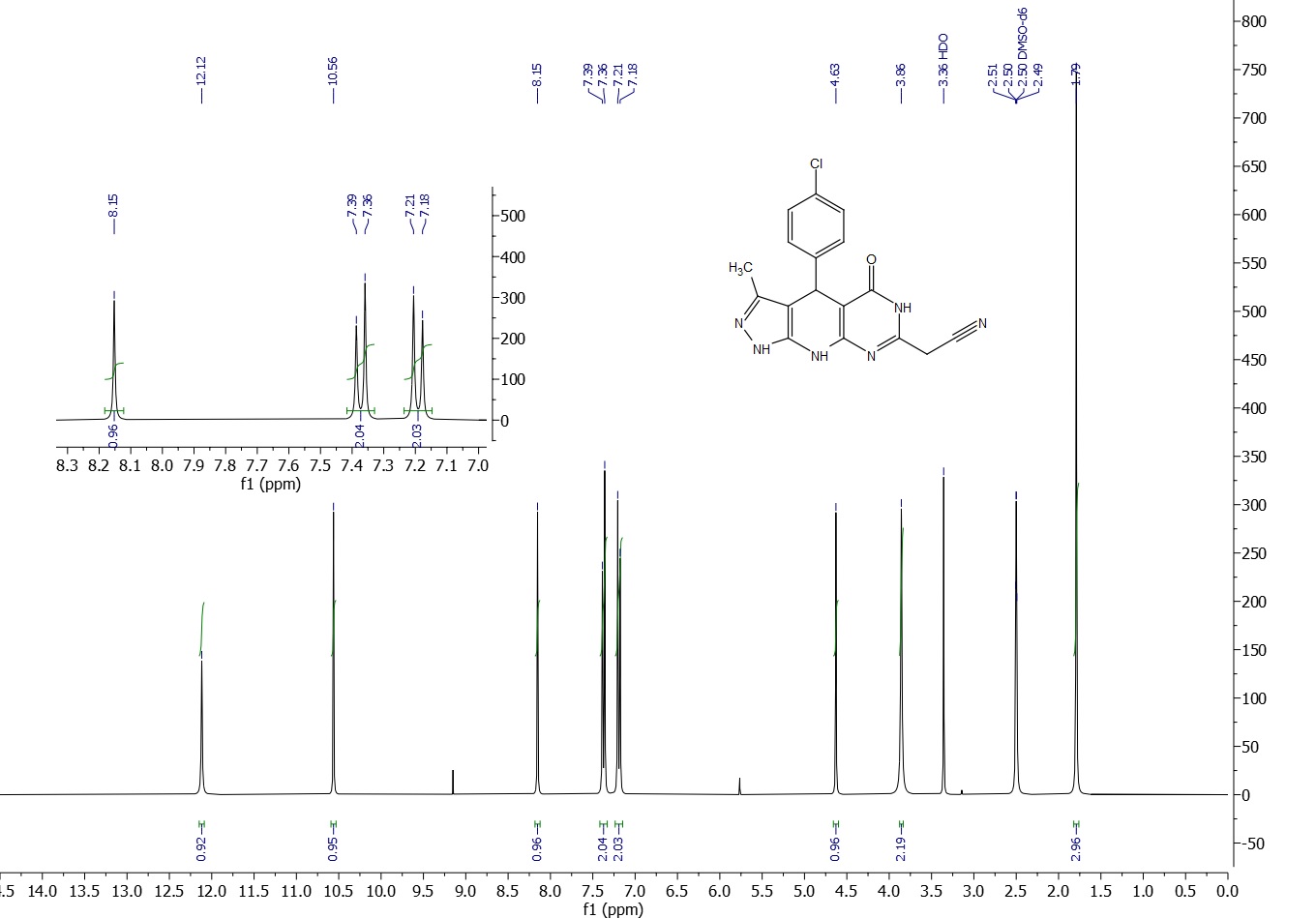

Supplement: Supplementary file 1 — Supplementary Material 1 [file 41598_2025_30217_MOESM1_ESM.docx]
